# Stratified assessment of platelets in sepsis: from dynamic counts to functional phenotypes

**DOI:** 10.3389/fimmu.2026.1783001

**Published:** 2026-04-10

**Authors:** Yafei Liu, Yanan Zou, Xiangtian Liu, Meifeng Li

**Affiliations:** 1 The Second Clinical Medical College of Shandong University of Medicine, Yantai, Shandong, China; 2 Department of Critical Care Medicine, Yantai Yuhuangding Hospital Affiliated to Qingdao University, Yantai, Shandong, China

**Keywords:** biomarkers, immune–coagulation regulation, platelet functional phenotypes, platelets, risk stratification, sepsis, therapeutic strategies

## Abstract

Sepsis is a major global cause of critical illness with high mortality. Sepsis-associated thrombocytopenia (SAT) is linked to markedly increased death risk and is a key indicator of poor prognosis. Although platelets are recognized as central players in the “inflammation–coagulation–immunity” network, most studies emphasize platelet counts rather than functional heterogeneity and underlying regulatory mechanisms, limiting the development of specific biomarkers and targeted therapies. Here, we characterize the “double-edged” role of platelets in sepsis. On the one hand, platelets recognize pathogens through pattern recognition receptors and exert anti-infective host defense functions; on the other hand, excessive platelet activation promotes endothelial injury and microthrombus formation through multiple signaling pathways and mediator release. These findings provide a concise framework linking platelet quantity, function, and mechanism in sepsis, and support the development of improved diagnostic and targeted treatment strategies.

## 1 Introduction

The classical functions of platelets—hemostasis and thrombosis—have been extensively elucidated over the past century. Recent research, however, has demonstrated that platelets are not merely “passive anucleate fragments,” but rather active circulating “immune sentinels” endowed with immune recognition and regulatory capabilities (1, 2). Platelets express Toll-like receptors (TLRs), NOD-like receptors (NLRs), and various adhesion/co-stimulatory molecules (3, 4), which enable them to recognize Pathogen-Associated Molecular Patterns (PAMPs) and Damage-Associated Molecular Patterns (DAMPs). Through signaling pathways such as Myeloid Differentiation Primary Response 88 (MyD88), G Protein-Coupled Receptors (GPCRs), and Immunoreceptor Tyrosine-Based Activation Motif/Hemi-Immunoreceptor Tyrosine-Based Activation Motif (ITAM/HemITAM) (5, 6), platelets integrate immune and coagulation signals, playing pivotal roles in inflammation regulation, immune cell recruitment, and microthrombosis formation (7). Thus, platelets are considered key nodes in the thrombo-inflammatory/immunothrombosis network.

In sepsis, this dual function is particularly critical. Clinical studies have indicated that sepsis patients often experience a decrease in platelet count and a remodeling of platelet functional phenotypes, with the degree of these alterations correlating with organ dysfunction and mortality risk (8, 9). However, clinical decision-making is often dominated by “count thresholds,” with insufficient attention paid to the dynamic changes in platelet activation, immune responses, and the coupling of coagulation-inflammation states. As a result, there is uncertainty surrounding interventions such as platelet transfusions, antiplatelet therapies, or platelet-stimulation therapies (4, 10, 11). More importantly, growing evidence suggests that the “immune-thrombosis inflammation” effects of platelets are context-dependent: during the early stages of sepsis, platelets may aid in pathogen limitation and endothelial homeostasis, while in sustained inflammation or microenvironmental imbalance, they may shift towards driving the amplification of thrombo-inflammatory responses and microcirculatory dysfunction (4, 12). Therefore, understanding the diverse origins of platelet abnormalities at the mechanistic level, along with recognizing their protective and deleterious transitions at different stages of disease, is essential for rethinking the current quantity-centric assessment model and providing a theoretical foundation for future stratified research and intervention strategies (13).

In light of this, the present article views platelets in sepsis as an “immune-coagulation switch.” By integrating their multifaceted roles in immune, coagulation, hematopoietic, and endothelial regulation, we aim to emphasize the importance of platelet functional states and temporal dynamics. This approach encourages a shift in clinical practice from a unidimensional count-based assessment to a multidimensional, dynamic understanding. It seeks to address the limitations of count thresholds and to explain the heterogeneity of thrombocytopenia in sepsis (TCP), providing new insights for subsequent functional assessments and targeted mechanistic interventions (14, 15).

## 2 Molecular basis of platelet immune regulation

Platelets are no longer viewed as passive anucleate hemostatic fragments. Instead, through the coordinated actions of surface receptor networks, intracellular secretory mediators, and intercellular interactions, they form a central molecular basis for immune regulation (16–19). In sepsis, platelet receptor expression, signaling activity, and secretory functions are dynamically reshaped by the pathological microenvironment (20, 21). This plasticity enables platelets to act as immune sentinels that recognize pathogens and damage signals, while integrating immune and coagulation cues to regulate innate immune activation, inflammatory responses, and the transition to adaptive immunity (22–24). Consequently, platelets serve as a central hub in the inflammatory–coagulation–immune network of sepsis.

### 2.1 Surface receptor regulatory network: a core system for signal recognition and effector initiation

Platelets are equipped with two functionally complementary receptor systems on their surface: pattern recognition receptors (PRRs) and non-PRR functional receptors. Together, these systems provide the structural basis for pathogen/damage signal sensing, thrombosis, and inflammatory amplification in sepsis, and their dysregulation or remodeling directly influences platelet pathological effects (25–27).

#### 2.1.1 Pattern recognition receptors: a sensing system for pathogen and damage signals

Platelets express a variety of Toll-like receptors (TLRs), which form the first line of defense against infections and damage (28).Among them, TLR4 directly recognizes bacterial lipopolysaccharide (LPS), whereas TLR2 forms heterodimers with TLR1 or TLR6 to specifically detect bacterial lipopeptides such as Pam3CSK4 and Pam2CSK4. These interactions activate the MyD88/NF-κB pathway, thereby inducing platelet activation, degranulation, and platelet–leukocyte interactions, which promote inflammation and thrombosis. Notably, TLR4-mediated NF-κB signaling can also upregulate P2Y12 receptor expression, enhancing platelet sensitivity to ADP. This cross-amplification between PRRs and metabolic signaling receptors further strengthens platelet responsiveness to infectious stimuli (29, 30).

As cytosolic PRRs, the NLR family primarily functions through inflammasome formation (22, 31, 32). Platelet-expressed NLRP3 can assemble with the adaptor protein ASC and pro-caspase-1 to form a functional complex, which promotes the maturation and release of inflammatory cytokines such as IL-1β and IL-18 in response to bacterial products and oxidative stress (4, 33, 34). In sepsis, excessive activation of the NLRP3 inflammasome amplifies systemic inflammation and is closely associated with neutrophil extracellular trap (NET) formation, making it a key node linking immune and thrombo-inflammatory responses (35, 36).

C-type lectin-like receptor 2 (CLEC-2) is one of the most important calcium-independent PRRs on the platelet surface (37, 38). Upon ligand binding, the HemITAM motif in its cytoplasmic tail undergoes tyrosine phosphorylation, leading to recruitment and activation of the Syk–PLCγ2 cascade. This pathway is highly similar to that of the collagen receptor GPVI and represents a central route for potent platelet activation (37). CLEC-2 can directly recognize exogenous toxins, such as rhodocytin, and can also be activated during bacterial infection (37, 38). By binding to podoplanin, it promotes immunothrombosis; meanwhile, under specific inflammatory conditions, it also contributes to the maintenance of vascular integrity, highlighting its functional duality (39–41).

**Figure 1:**
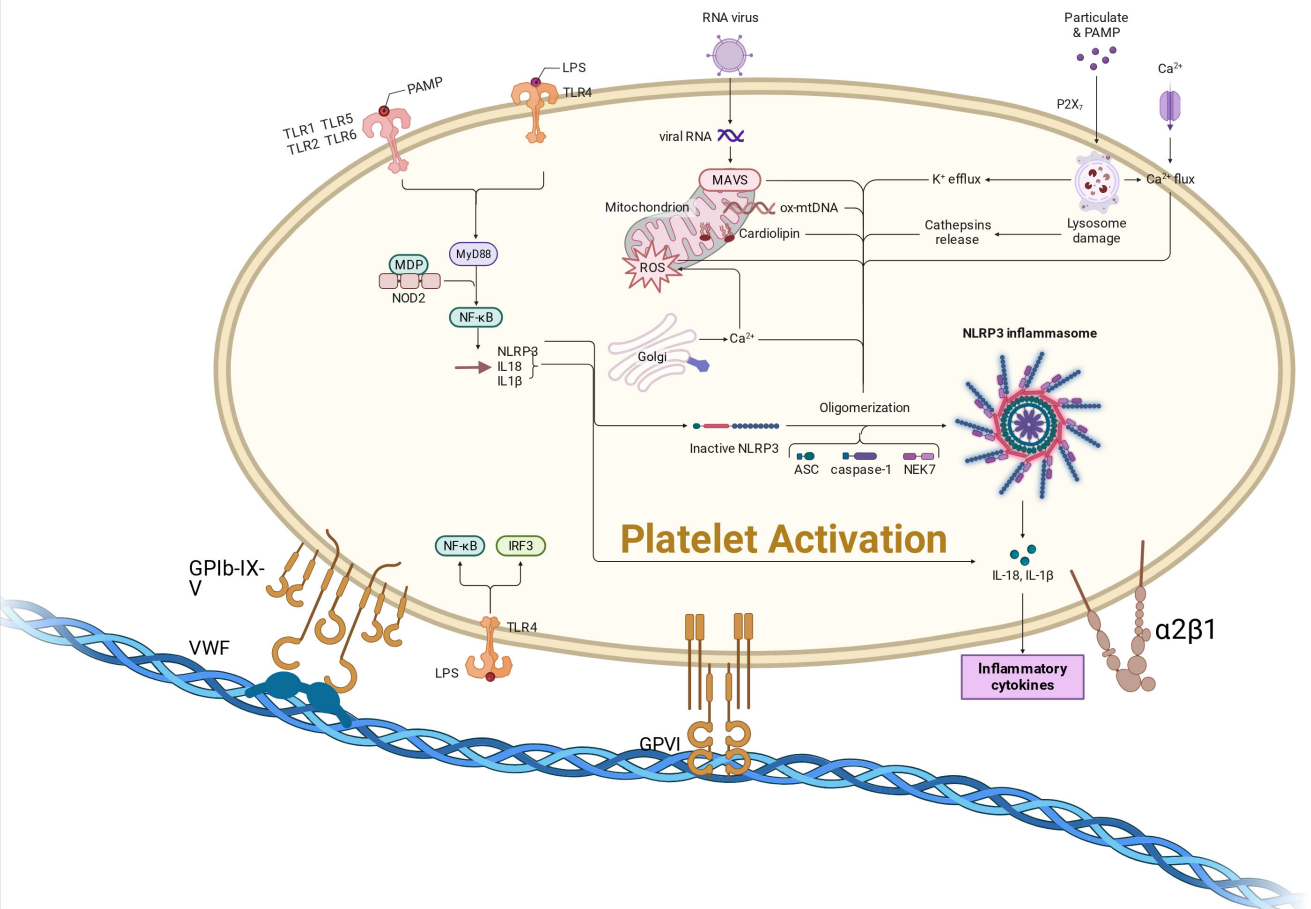
Activation of platelets by pathogens via toll-like receptors. The figure illustrates the mechanism by which pathogens activate platelets through Toll-like receptors (TLRs) and NOD-like receptors (NLRs). The Toll-like receptors (TLR2, TLR4, TLR9) expressed on the platelet surface recognize PAMPs or DAMPs, triggering immune activation pathways that lead to platelet activation, degranulation, and interactions with immune cells. In addition, NLR family receptors within platelets also play a role in regulating immune responses. NOD2 (NOD-Like Receptor 2) recognizes intracellular pathogens or damage signals, activating the NLRP3 inflammasome, thereby promoting the maturation and release of inflammatory cytokines such as IL-1β.

In summary, TLRs, the NLRP3 inflammasome, and CLEC-2 constitute a core platelet danger-sensing network in sepsis. TLRs initiate proinflammatory signaling (25), NLRP3 amplifies inflammatory responses (4, 42), and CLEC-2 drives immunothrombosis (37, 38). Together, they mediate the initial response to pathogen clearance and inflammatory regulation, as illustrated in **Figure 1**, whereas their dysregulated activation may further exacerbate the sustained amplification of immune-thromboinflammatory responses (43).

#### 2.1.2 Non-PRR functional receptors: an effector system for thrombo-inflammatory coupling

In addition to PRRs, platelets express adhesion and aggregation receptors, signaling receptors, and immune-related receptors. Although these receptors do not directly recognize pathogens, they can be hijacked by the inflammatory microenvironment in sepsis and become key mediators of thrombosis and inflammatory amplification (11, 25, 42, 43). Functionally, adhesion and aggregation receptors, such as integrin αIIbβ3 and GPIbα, bind ligands including fibrinogen and von Willebrand factor (vWF), thereby mediating interplatelet cross-linking and initial adhesion to the vascular wall and providing the physical basis for immunothrombosis (44). Signaling receptors, such as P2Y12 and PAR1/4, sense microenvironmental cues including ADP and thrombin, trigger intracellular cascades, and regulate platelet activation, degranulation, and thrombus stabilization (45). Immune-related receptors, such as FcγRIIa and CD47, directly connect platelets with immune cell interactions and participate in leukocyte recruitment, immune signal transmission, and immune homeostasis.

To clearly present the pathological significance and clinical implications of the core receptors, we summarize the key receptor-ligand/pathway interactions, sepsis relevance, clinical application value, and evidence levels in **Table 1**, which serves as a reference for subsequent mechanistic interpretations and clinical stratification.

**Table 1:** Key platelet receptors and their clinical significance in sepsis.

Receptor-ligand/pathway	Sepsis relevance	Clinical implication	Evidence level	References
TLR4–LPS/MyD88	Promotes activation, interaction with neutrophils, processes related to NETs (Neutrophil Extracellular Traps)	Immune thrombosis related	Human/Animal/*In vitro*	(8, 46)
TLR4-NF-κB/P65	Promotes activation of NF-κB/P65 pathway, upregulates platelet P2Y receptor expression, enhances platelet aggregation and activation	Immune thrombosis related	Human/Animal/*In vitro*	(45)
NLRP3inflammasome	IL-1β related inflammation amplification	Explains inflammation amplification/organ damage association	Animal/*In vitro*	(4, 8)
CLEC-2–hemITAM–Syk phosphorylation	Immune thrombosis and endothelial related	Immune thrombosis related	Animal/*In vitro*	(44, 47, 48)
P-selectin(CD62P)–PSGL-1	Formation of PLA (Platelet-Leukocyte Aggregates)/PNCs (Platelet-Neutrophil Complexes)	Strength of immune-coagulation coupling	Human/Animal/*In vitro*	(4, 49–51)
Integrin αIIbβ3 (αIIbβ3)	Aggregation/thrombosis stabilization, outside-in signaling	High activation vs. exhaustion phenotype	Human/Animal	(52, 53)
GPVI(Collagen/Fibrinogen)	Collagen-dependent response defect/receptor shedding	Explains differing reactivity in same platelet count	Human/Animal/*In vitro*	(15, 44, 54)
Glycoprotein Ibα (GPIbα)–vWF	Initial adhesion, receptor shedding affects adhesion/interactions	Microcirculatory adhesion levels	Human/*In vitro*	(55, 56)
P2Y12 (P2Y12 Receptor)–ADP	Response threshold and aggregation stability; can be high reactive or exhausted	Explains differing reactivity in same platelet count	Human/Animal/*In vitro*	(30, 57)
Protease-Activated Receptor 1/4 (Thrombin - PAR1/4)–G-protein coupled pathways	Strong activation and inflammation coupling	Exhaustion/high activation background	Human/Animal/*In vitro*	(8, 58–60)
Fcγ Receptor IIa (FcγRIIa) – Syk/Src	Immune complex related activation/destruction	Alerts to potential immune-mediated destruction	Human/Animal/*In vitro*	(61–63)
CD47–Signal Regulatory Protein α (SIRPα)	“Brake” mechanism on monocyte inflammatory response	Thrombocytopenia may release homeostatic brake	Animal/*In vitro*/Human	(64)

At present, these receptors can be quantitatively assessed using combined methodological approaches. Flow cytometry can simultaneously capture surface receptor abundance, activation markers, and intracellular signaling molecules (65). Western blotting and co-immunoprecipitation can verify intracellular signaling events and protein complex formation (66). Platelet aggregometry and microfluidic chips can evaluate receptor-mediated aggregation and adhesion functions (67, 68). ELISA and multiplex bead-based assays can detect the release of related inflammatory mediators (69). However, these techniques remain limited by procedural complexity, high cost, and the lack of integrated analytical frameworks, which restricts their translation into clinically practical assessment systems (65).

### 2.2 Intracellular secretory granules: mediators of signal amplification and paracrine regulation

**Figure 2:**
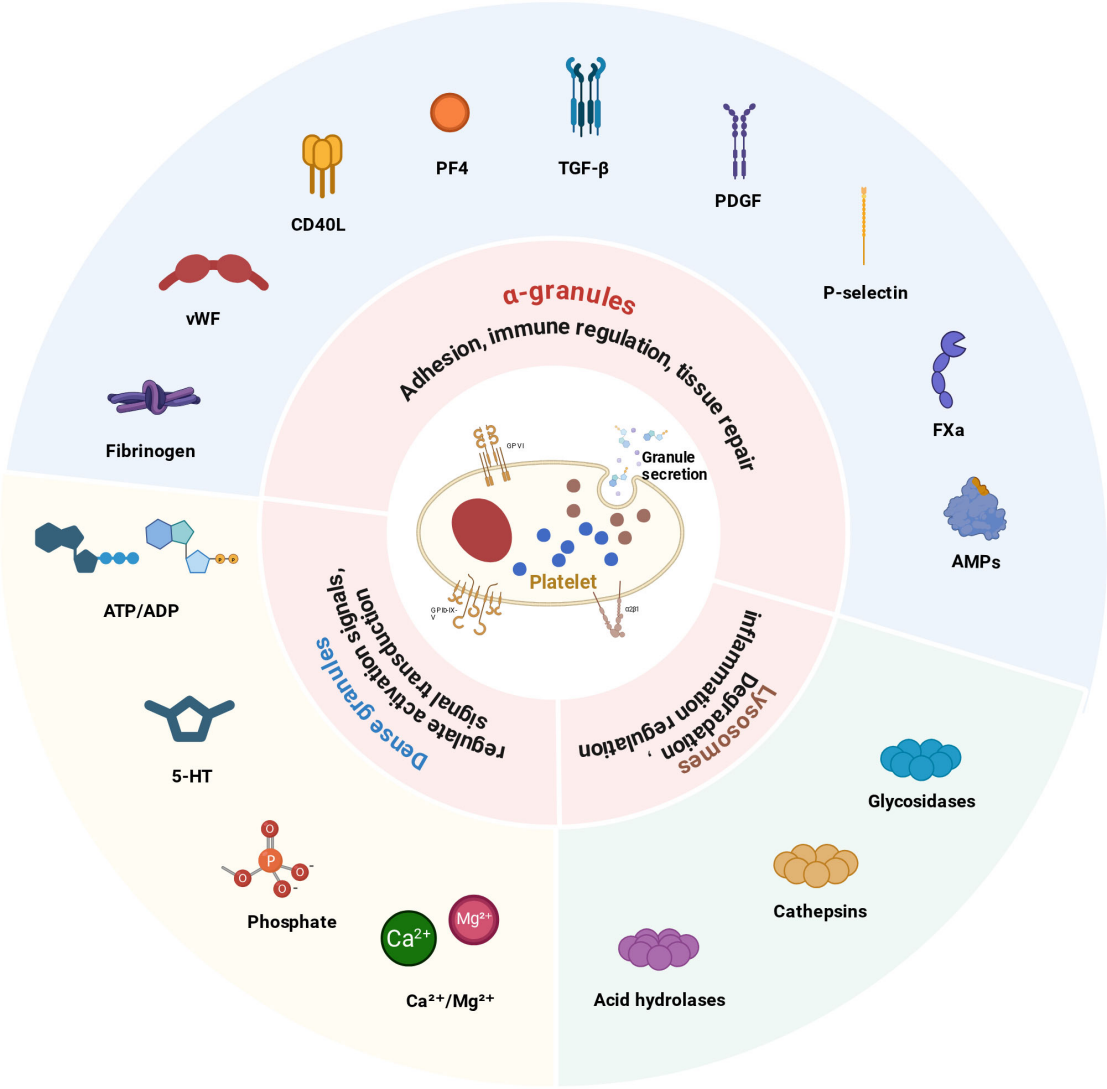
Platelet intracellular granules and their functions. The figure illustrates the key bioactive molecules stored in the three types of platelet secretory granules and their associated functions.α-granules: fibrinogen; von Willebrand factor (vWF); CD40L; platelet factor 4 (PF4); transforming growth factor β (TGF-β); platelet-derived growth factor (PDGF); P-selectin; coagulation factor Xa (FXa); antimicrobial peptides (AMPs).Dense granules: ATP/ADP (Adenosine Triphosphate/Adenosine Diphosphate); 5-hydroxytryptamine (5-HT); phosphates; Ca²^+^/Mg²^+^ (calcium/magnesium ions).Lysosomes: glycosidases; cathepsins; acid hydrolases.

Platelets release a variety of bioactive molecules through the intracellular storage of three types of secretory granules—α-granules, dense granules, and lysosomes. This release constitutes the primary effector pathway through which platelets mediate immune and inflammation regulation (70, 71).Notably, mediator release is highly context-dependent: Early in the disease, the release of antimicrobial molecules and chemokines may help limit pathogens and facilitate local defense; however, in the context of sustained inflammation or dysregulated control, excessive release of pro-inflammatory cytokines and coagulation-related molecules may drive the amplification of systemic inflammatory responses and the onset of microcirculatory dysfunction (72, 73). As the most abundant secretory granules, α-granules store key molecules such as fibrinogen, von Willebrand factor (vWF), CD40L, platelet factor 4 (PF4), and transforming growth factor-β (TGF-β). Among them, chemokines such as PF4 and CXCL7 recruit immune cells (17, 71), whereas vWF and fibrinogen enhance platelet adhesion and thrombus formation (74, 75). Dense granules mainly release signaling molecules such as ATP/ADP and serotonin (5-HT), the latter of which is involved in vascular tone regulation and inflammatory responses (76). Lysosomes release acidic hydrolases, including glycosidases and cathepsins, which contribute to tissue remodeling and inflammatory regulation (70, 77). The three major types of intracellular platelet granules and their principal functions are summarized in **Figure 2**, which provides a reference for subsequent mechanistic interpretation and clinical stratification.

In addition to granule-mediated secretion, platelets can further expand their secretory functions through the release of extracellular vesicles (78). Extracellular vesicles have become a major focus in life science research in recent years, and the unique roles of platelet-derived extracellular vesicles (PEVs) in immune and inflammatory regulation are being increasingly clarified. PEVs are enriched in phosphatidylserine, inflammatory mediators, and adhesion molecules. Unlike intracellular granules, they originate from intraluminal vesicles released after fusion of multivesicular bodies with the plasma membrane, and they function primarily through intercellular signal transfer rather than direct release of granule contents. Important roles for PEVs have been identified in inflammatory regulation, tumor progression, and regenerative medicine (78–81). In sepsis, PEV levels are markedly increased and can amplify thrombo-inflammatory responses by promoting platelet–endothelial adhesion and neutrophil/NET formation. Combined with lactate, PEVs may also improve the sensitivity for predicting acute kidney injury (80, 82, 83).Currently, soluble mediators such as PF4, CD40L, and IL-8 can be quantified by ELISA or multiplex bead-based assays. Proteomic approaches can be used to analyze differences in secretory profiles among platelet functional phenotypes, whereas nanoparticle tracking analysis (NTA) combined with Western blotting can assess PEV concentration, size distribution, and the expression of marker proteins such as CD41 and CD61 (81, 84). These methods provide important technical support for investigating the regulatory mechanisms of platelet secretion and clarifying its role in inflammation-related diseases, while also laying a foundation for future diagnostic and therapeutic target discovery.

We summarize the three types of intracellular platelet granules and their primary functions in **Figure 2** which provides a reference for subsequent mechanistic interpretation and clinical stratification.

### 2.3 Platelets and immune cells: connections with neutrophils, monocytes/macrophages, and adaptive immunity

**Figure 3:**
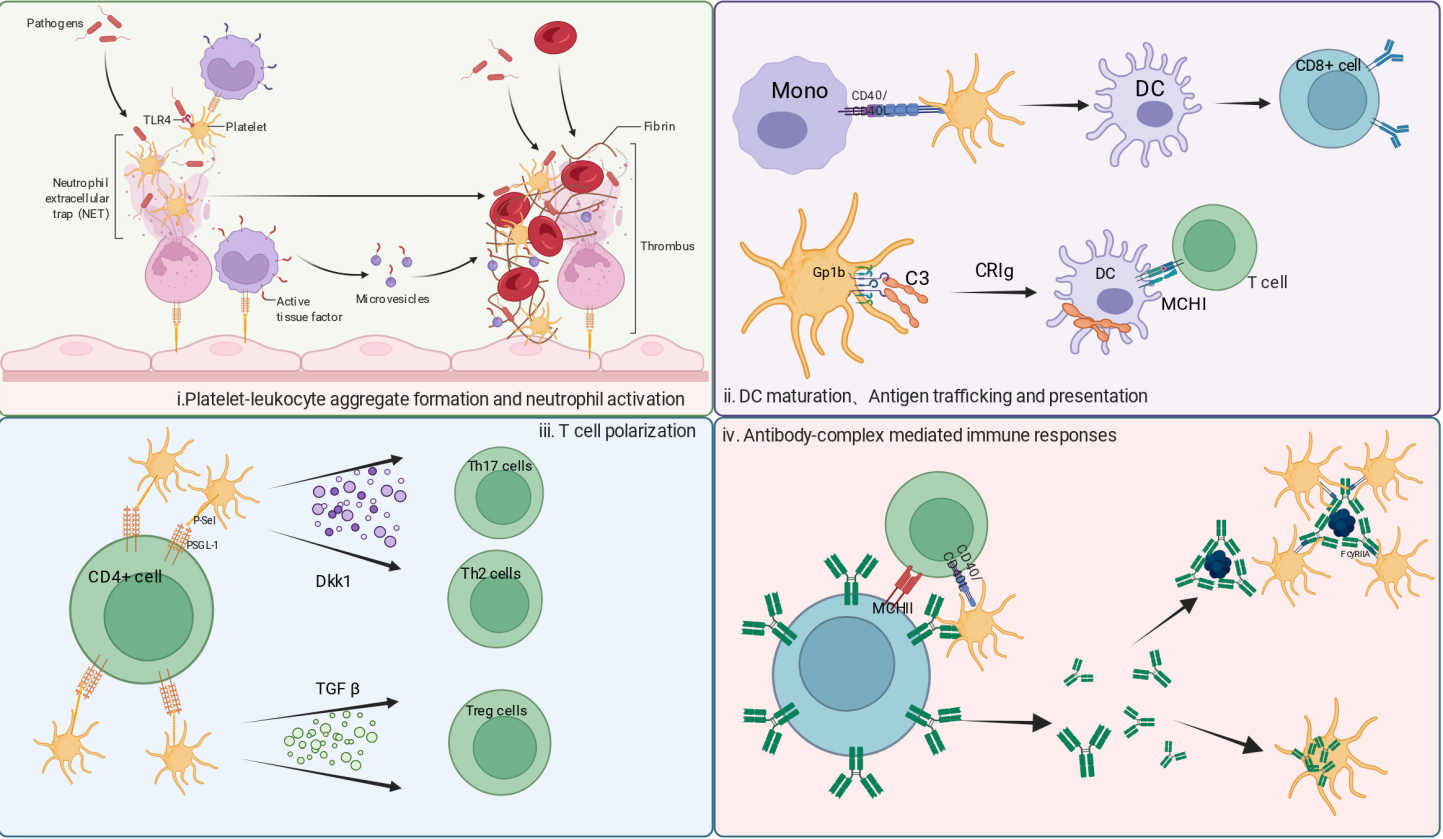
Interaction between platelets and immune cells. i:Platelet Regulation of Innate Immune Response: Platelets interact with neutrophils, promoting the formation of platelet-leukocyte aggregates and activating neutrophils for immune responses. The interaction between platelets and neutrophils can intensify the inflammatory response and contribute to microthrombosis formation.ii: Dendritic Cell Maturation, Antigen Presentation, and T Cell Activation: Platelets interact with dendritic cells, promoting their maturation and antigen-presenting functions, which in turn activate T cells.iii: T Cell Polarization: Platelets regulate T cell differentiation into pro-inflammatory (e.g., Th1/Th17) or regulatory (Treg) subsets by secreting factors such as CD40L and Dkk1, thus balancing the immune response.iv: Antibody-Immune Complex Mediated Immune Response: Storage and Release of Antibodies: Platelets absorb pre-existing antibodies from circulation and release them at the site of pathogen infection, enhancing local humoral immunity. Immune Complex Recognition: Platelets bind antibody-antigen complexes via FcγRIIA, promoting pathogen opsonization and phagocytic clearance (e.g., antiviral and antibacterial immunity).

In addition, platelets serve as a key “hub” connecting innate immunity and adaptive immunity. Recent studies have revealed that platelets, through specific receptor-ligand interactions, regulate both innate and adaptive immunity, establishing a sophisticated bidirectional regulatory network (**Figure 3**). This cooperative effect goes far beyond simple physical aggregation.

In innate immune regulation, platelet surface P-selectin (CD62P) binds to PSGL-1 on neutrophils to initiate adhesion and rolling, followed by ICAM2–LFA1 engagement and β2 integrin activation, which further strengthen adhesion and promote neutrophil transendothelial migration (49). Meanwhile, platelets can induce NETosis through the TLR4–P-selectin axis and release mediators such as platelet-activating factor (PAF), thromboxane A2 (TXA2), and interleukin-8 (IL-8), thereby enhancing neutrophil phagocytic and bactericidal activity while limiting excessive inflammatory injury (85). However, excessive NET formation can aggravate tissue damage and immunothrombosis (4, 50).For macrophages, platelets can specifically activate the surface G protein-coupled receptor 35 (GPR35) on macrophages, enhancing their phagocytosis and inflammatory responses. Additionally, through surface CLEC-2, platelets form an “immune synapse” with inflammatory macrophage podoplanin and macrophage antigen-1 (Mac-1), playing a core role in limiting bacterial spread and maintaining tissue homeostasis (4, 41).

In the context of antigen presentation and the connection to adaptive immunity, platelets and platelet-derived extracellular vesicles (PEVs) capture pathogens or self-antigens, which are then transported via the lymphatic circulation to secondary lymphoid organs (86). The surface expression of Major Histocompatibility Complex Class I (MHC-I), CD40 Ligand (CD40L), and CD86 on platelets directly participates in antigen presentation (87). Furthermore, the CD40-CD40L signaling pathway promotes dendritic cell (DC) maturation and the secretion of pro-inflammatory cytokines, optimizing antigen presentation efficiency (88). Through GPIbα-mediated binding to opsonized pathogens, platelets further facilitate antigen delivery, while CD40L promotes Th1/Th17 differentiation (86). secrete TGF-β and Dkk1 to induce regulatory T cells (Tregs), and enhance antibody-dependent cellular cytotoxicity (ADCC) via FcγRIIA binding to immune complexes. These interactions can be evaluated by flow cytometric analysis of platelet–leukocyte aggregates (PLAs/PNCs), immunofluorescence staining combined with ELISA for NET-related markers, and co-culture systems assessing DC maturation markers and T-cell polarization subsets. Together, these approaches provide direct experimental evidence for platelet-mediated immune regulation (89, 90).

### 2.4 Platelet heterogeneity: the basis of functional specialization and phenotypic diversity

Circulating platelets are not a functionally uniform population, but instead exhibit marked structural and functional heterogeneity. This heterogeneity spans the entire platelet life cycle, from hematopoietic stem cell differentiation to platelet clearance, and provides an essential basis for adaptation to different pathological microenvironments (91, 92).

Platelet heterogeneity arises from the combined effects of intrinsic and extrinsic factors. Intrinsic factors include genetic and epigenetic background, megakaryocyte differentiation trajectories, and asymmetric material allocation during thrombopoiesis. Extrinsic factors include circulating agonists and inhibitory signals, interactions with other cells, inflammatory or infectious states, and drug exposure (91–93). Among these, the tissue origin and differentiation pathway of megakaryocytes represent key upstream determinants of platelet heterogeneity. Under homeostatic conditions, the bone marrow is the principal site of platelet production, where megakaryocytes are highly polyploid (up to 64N or more) and specialized for efficient platelet generation (94, 95). In contrast, lung megakaryocytes are often low-ploidy (2N), express high levels of immune-related molecules such as major histocompatibility complex class II (MHC II) and intercellular adhesion molecule 1 (ICAM1), and possess antigen uptake and presentation capacity. During inflammation or infection, they can markedly increase platelet production; under steady-state conditions, approximately 5%–10% of circulating platelets originate from the lung, and this proportion rises significantly during thrombocytopenia (92, 96, 97). In the middle to late stages of sepsis, the spleen may become a major site of platelet production, and spleen-derived platelets express higher levels of CD40 ligand, indicating stronger immunoregulatory potential (9).

In addition, megakaryocyte differentiation follows two major routes. The classical Flk2/Flt3-dependent pathway generates platelets through stepwise differentiation from multipotent progenitors (98). By contrast, in the Flk2/Flt3-independent bypass pathway, platelet-biased hematopoietic stem cells can directly differentiate into megakaryocyte progenitors, rapidly producing platelets with high reactivity and immune-related features; this pathway expands markedly under stress conditions such as infection (99, 100). Although platelets are anucleate, they retain precursor mRNAs and a functional translational machinery, enabling dynamic regulation of protein synthesis in response to environmental stimuli and further amplifying functional diversity (92). Based on circulating age, functional phenotype, and origin/differentiation pathway, platelets can be classified into distinct subpopulations with specific biological characteristics. Details of these subpopulations are provided in **Table 2**.

**Table 2:** Dominant mechanisms, clinical features, and potential intervention directions of abnormal platelet subtypes.

Classification dimension	Subtype	Key stage of differentiation	Major driving factors
Circulatory age	Reticulated platelets (RPs)	Early after release from mature megakaryocytes into the circulation (within 24–48 h of generation)	Activation of classical or emergency megakaryopoiesis in the bone marrow; progenitor-to-megakaryocyte differentiation; regulation of direct or stepwise differentiation from hematopoietic stem cells; compensatory hematopoiesis in response to inflammatory stress
	Aged platelets	Progressive aging during circulation (7–10 days in humans; 4–5 days in mice), with gradual initiation of apoptotic programs	Time-dependent activation of platelet apoptotic programs; enhanced aging-related direct differentiation pathways of hematopoietic stem cells in older individuals; aging-associated remodeling of megakaryocyte gene expression, including pathways related to mitochondrial function, oxidative stress, and immune signaling
Functional phenotype	Aggregatory platelets	Immediate activation after vascular injury (within minutes), or activation in circulation under low-intensity stimulation (e.g., ADP, collagen)	Low-intensity stimuli such as ADP and collagen; platelet priming mediated by activating proteins such as von Willebrand factor (vWF); intercellular signaling through P-selectin/PSGL-1 and GPIbα/CD11b
	Procoagulant platelets	After dual strong stimulation (e.g., collagen + thrombin, S100A8/9); typically during the cytokine storm stage of sepsis (24–72 h after onset)	Dual strong stimulation by collagen plus thrombin or S100A8/9; supramaximal calcium signaling mediated by the mitochondrial permeability transition pore and calcium uniporter; FcγRIIA activation; signaling pathways promoting phosphatidylserine exposure and thromboxane generation
	Immune-secretory platelets	Rapid degranulation after activation (within minutes); persist from early to middle stages of sepsis and during infection/inflammation onset; also generated during splenic or pulmonary thrombopoiesis	Infection- and damage-related signals (PAMPs/DAMPs) under inflammatory conditions such as sepsis; immunoregulatory drive through CD40–CD40L signaling; noncanonical TGF-β receptor signaling mediated by platelet-derived β2-microglobulin (β2M); immune differentiation programming of emergency megakaryocytes in the lung and spleen; degranulation signaling activated in the inflammatory microenvironment
Origin/differentiation pathway	Bone marrow-derived platelets	Continuous production under homeostasis, with compensatory increase during early inflammation	Classical bone marrow megakaryopoiesis under homeostatic conditions; basal regulation by Lepr^+^ stromal cells and CD51^+^PDGFRα^+^ pericytes in the bone marrow; compensatory thrombopoietic signaling during early inflammation; baseline regulation of direct or stepwise differentiation from hematopoietic stem cells
	Lung-derived platelets	Under emergency conditions (e.g., infection, thrombosis), after activation of pulmonary megakaryocytes	Activation of pulmonary megakaryocytes by emergency stimuli such as infection or thrombosis; immune differentiation programming of pulmonary megakaryocytes; activation of immunoregulatory megakaryocyte subsets in distant organs during inflammation; PAMP/DAMP-mediated emergency thrombopoietic signaling in the lung
	Spleen-derived platelets	Middle to late stages of sepsis (3–7 days after onset), when the spleen becomes a major site of platelet production	Systemic inflammatory stress signals in the middle to late stages of sepsis; adrenergic signaling-mediated migration of bone marrow megakaryocyte–erythroid progenitors to the spleen; IL-3-induced splenic megakaryocyte maturation; functional activation signals in splenic megakaryocytes during infectious stress

Different platelet subpopulations can be identified by flow cytometry through assessment of surface markers such as CD40L, CD47, and GPVI, activation markers including CD62P and PAC-1, and phosphatidylserine (PS) exposure. These analyses can be combined with morphological parameters such as mean platelet volume (MPV) and immature platelet fraction (IPF), as well as platelet aggregometry to evaluate responsiveness to ADP and collagen, thereby providing key technical support for precise clinical stratification (101–104).

### 2.5 Crosstalk networks in immune regulation: from resting state to functional phenotype switching

Platelet-mediated immune regulation is a systemic process involving coordinated receptor activation, signal crosstalk, and dynamic phenotypic differentiation. Through the integration of multiple signaling pathways, this network drives the transition of platelets from a resting state to hyperreactive and ultimately exhausted states, thereby determining their dual protective and deleterious roles in sepsis.

PAMPs and DAMPs can simultaneously trigger the coordinated activation of multiple platelet surface receptors. After recognizing PAMPs such as lipopolysaccharide (LPS), TLR2/4 activate the MyD88/NF-κB pathway (105, 106), whereas CLEC-2 initiates the Syk–PLCγ2 cascade through its HemITAM motif (41), and GPIbα mediates adhesive signaling through binding to von Willebrand factor (vWF). These receptor systems converge on shared signaling mediators, including Syk and PLCγ, thereby enhancing platelet sensitivity and responsiveness to infectious or injury-related signals. At the same time, platelet heterogeneity provides the basis for functional specialization of receptor crosstalk. For example, lung-derived platelets express higher levels of immune-related receptors, whereas reticulated platelets are more reactive, resulting in subtype-specific differences in coordinated activation (8, 107).

At the intracellular level, upstream receptor signals are finely tuned through multiple crosstalk mechanisms, forming a multilayered regulatory network centered on calcium signaling, granule–organelle coupling, and kinase pathway interactions. Dynamic signaling interplay and functional antagonism together allow precise control of platelet activation. Notably, GPIbα is not limited to mediating initial platelet adhesion to the vascular wall. Its cytoplasmic tail can regulate PKCα activity through binding to 14-3-3ζ, thereby promoting integrin αIIbβ3 activation, granule secretion, and phosphatidylserine (PS) exposure, and thus serving as a key node linking adhesion signals to global platelet activation (55). Calcium signaling is a central hub of platelet activation: store-operated calcium entry (SOCE), mediated by STIM and Orai, directly regulates platelet aggregation and granule secretion, whereas mitochondria modulate intracellular Ca2+ homeostasis and reactive oxygen species (ROS) generation, thereby influencing ITAM pathway activity and the phosphorylation of Syk and PLCγ2. This coupling links organelle function to classical immune signaling pathways (108–110). In addition, the interaction between ion homeostasis and kinase signaling further increases network complexity. MAGT1 deficiency causes magnesium imbalance, which enhances Syk–LAT–PLCγ2 phosphorylation in the GPVI pathway while weakening PKC-mediated inhibitory circuits, ultimately increasing calcium influx and platelet hyperreactivity. In this context, TRPC6 acts as an important mediator linking magnesium homeostasis to platelet activation (109).

The integration and tuning of upstream signaling networks ultimately drive platelets toward three major functional phenotypes, the relative proportions of which are dynamically reshaped by the septic microenvironment and directly reflect platelet functional switching. First, the adhesive–aggregatory phenotype is centered on integrin αIIbβ3 activation and mediates interplatelet cross-linking and platelet adhesion to the vascular wall, thereby providing a physical barrier for early pathogen containment. Second, the inflammatory secretory phenotype is characterized by high expression of CD62P and CD40L and promotes immune cell recruitment through the release of mediators such as PF4 and IL-8, thereby strengthening local inflammatory defense. Third, the procoagulant phenotype depends on PS exposure and ANO6 channel activation, accelerating thrombin generation and microthrombus formation. This phenotype may be protective during infection containment, but excessive activation can lead to widespread microvascular thrombosis and impaired tissue perfusion.

Platelets from different origins have intrinsically distinct thresholds for signal responsiveness, which in turn determine the efficiency of phenotype switching and the magnitude of pathological effects (107, 111). A deeper understanding of this signaling network may provide a theoretical basis for identifying the protective or deleterious state of platelets through functional phenotyping, while also offering potential targets for stratified intervention (112).

## 3 Platelet immune effects in sepsis: protective roles and harmful mechanisms

The immune effects of platelets in sepsis are highly context-dependent and functionally bidirectional, mainly reflecting a dynamic balance among three core functional modules: anti-infective defense in the early stage, immunothrombosis-driven microcirculatory injury after inflammatory dysregulation, and immune homeostasis throughout the disease course. These three modules do not replace one another linearly but instead dynamically overlap and shift in weight as the septic microenvironment changes and as the disease progresses (113, 114). In the early stages upon admission to the Intensive Care Unit (ICU), platelets predominantly mediate defensive immune effects (9, 115). As inflammation persists and endothelial injury progresses, they gradually transition to a pathological stage driven by immune thrombosis and related damage (11, 116, 117). In the later stages of the disease, an interplay between immune suppression and platelet functional exhaustion may emerge (118, 119).This functional transition reflects adaptive platelet remodeling in response to the septic microenvironment and is primarily determined by inflammatory intensity, signaling network activation thresholds, and platelet subset composition.

### 3.1 Early infection: platelet involvement in pathogen limitation and immune cell recruitment

In the early stages of sepsis or focal infection, platelets precisely sense PAMPs and DAMPs through PRRs, initiating targeted local defense responses. On the one hand, platelets quickly adhere and aggregate to form a physical barrier, working with phagocytes to precisely localize and engulf pathogens (120). On the other hand, they release antimicrobial peptides (AMPs), chemokines (e.g., IL-8), and growth factors from α-granules and dense granules, significantly enhancing immune cell recruitment and activation (121).

Importantly, platelets are derived exclusively from megakaryocytes, and megakaryopoiesis is strongly context-dependent. Under homeostatic conditions, megakaryocytes are generated only in the bone marrow, where their high ploidy status (up to 64N or more) supports efficient platelet production and makes the bone marrow the principal source of circulating platelets (122, 123). Under stress conditions such as inflammation or infection, bone marrow thrombopoiesis remains the basal source, while the spleen and lung initiate emergency megakaryopoiesis and serve as supplementary sources; other organs do not have the capacity to generate megakaryocytes or platelets (97). Lung megakaryocytes are generally low-ploidy (2N), express high levels of immune-related molecules such as major histocompatibility complex class II (MHC II) and intercellular adhesion molecule 1 (ICAM1), and have antigen uptake and presentation capacity. Under steady-state conditions, approximately 5%–10% of circulating platelets are derived from the lung, and this proportion can increase markedly in early sepsis. These platelets appear to have particular advantages in immune recognition and regulation (92, 97, 124). The spleen, in turn, activates an emergency hematopoietic program during sepsis. Megakaryocyte–erythroid progenitors from the bone marrow migrate to the spleen under adrenergic signaling and differentiate into functionally mature megakaryocytes. The resulting post-sepsis platelets express high levels of CD40 ligand and display stronger immune-enhancing properties. Animal studies have shown that transfusion of these platelets significantly reduces mortality in sepsis models, supporting their key role in early immune defense (9).

The early protective effects of platelets depend on threshold-controlled moderate activation. Within a physiological range, platelet activation enhances local defense; however, excessive or sustained activation may exceed this defensive threshold and initiate the pathological cascade of immunothrombosis (4, 115). Therefore, interventions targeting platelet activity in the early stages of sepsis must be approached cautiously. Premature interventions could lead to a weakening of defense functions (referred to as “miswindow inhibition”), which could compromise the full expression of platelet-mediated early anti-infection effects.

### 3.2 Dysregulated stage: immunothrombosis and microcirculatory injury

As inflammation continues, endothelial injury occurs, and coagulation system abnormalities activate in sepsis, platelet functional phenotypes undergo pathological changes, initiating the “immune thrombosis-mediated pathological damage amplification” pathway (12, 61).The core mechanism underlying this process is the synergistic amplification of platelet–immune cell interactions and microthrombus deposition. Platelets interact with neutrophils and monocytes via receptor-ligand axes such as P-selectin (CD62P)/P-selectin glycoprotein ligand-1 (PSGL-1), forming platelet-leukocyte aggregates (PLA/PNCs), and inducing NETs release (12, 61). Simultaneously, coagulation-related molecules (e.g., tissue factor, vWF) and inflammatory mediators released by activated platelets promote the deposition of fibrin-platelet microthrombi within the microcirculation, resulting in tissue perfusion disturbances and the progression of organ dysfunction (116, 125, 126).

At this stage, platelets move beyond their role in coagulation, becoming key structural factors that anchor immune signals to the microvascular wall. Abnormal platelet activation can exacerbate the persistence and amplification of local inflammation. It is noteworthy that activation of this pathological module does not necessarily rely on a significant decrease in platelet count: even if platelet counts remain normal, high-reactivity platelet activation with strong coupling between inflammation and coagulation systems indicates an elevated risk of organ dysfunction (10, 30, 126, 127). In clinical settings, when patients present with “normal platelet counts but insufficient tissue perfusion, elevated blood lactate, or abnormal coagulation parameters,” immune thrombosis-mediated pathological damage is often the core driving factor of disease progression, which cannot be explained by platelet count alone (128, 129).These findings further support the greater clinical value of platelet functional assessment over platelet count alone in sepsis.

### 3.3 Regulation throughout the disease course: platelet-mediated maintenance of immune homeostasis

**Figure 4:**
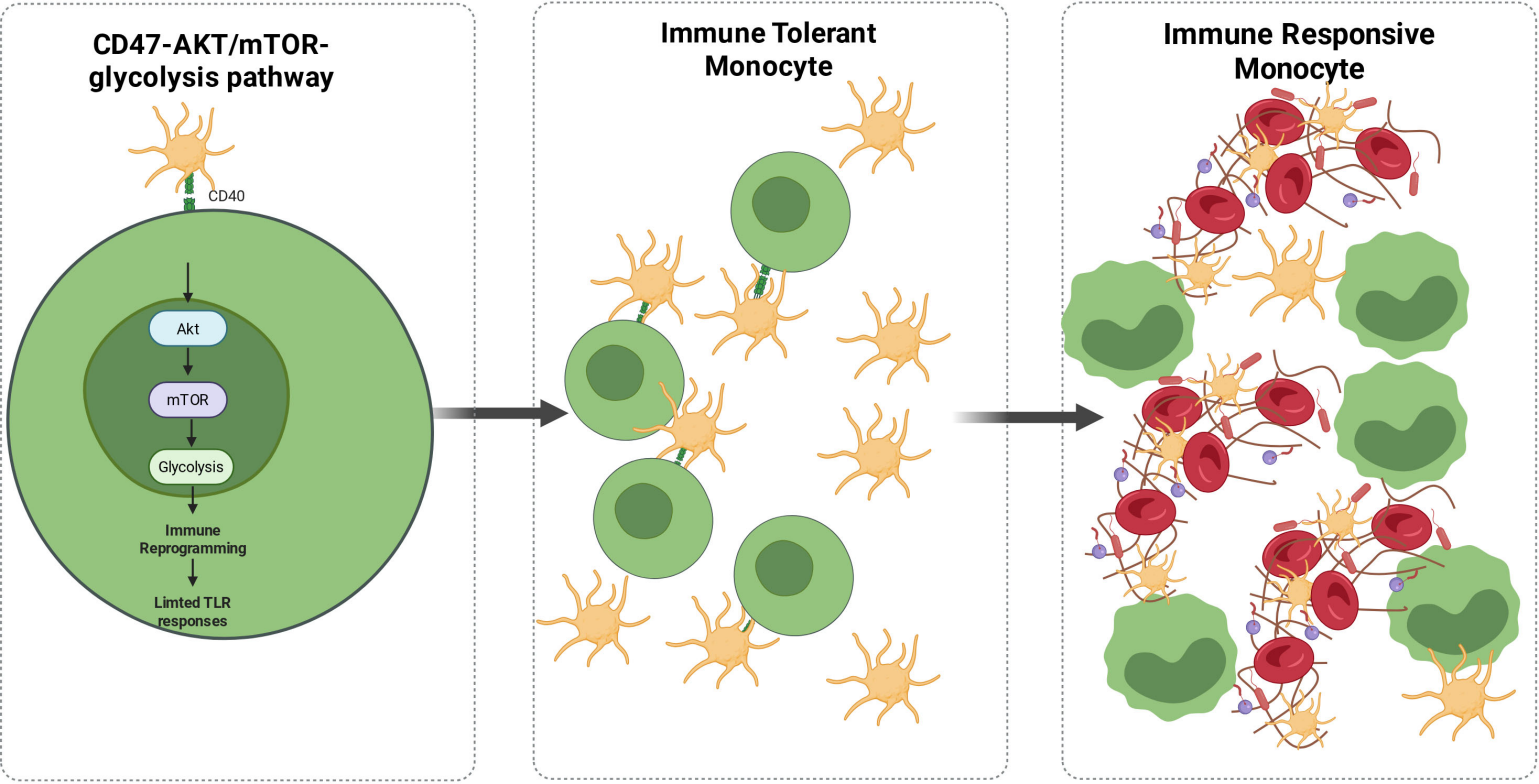
Platelet-mediated immunosuppressive effect on monocytes. In sepsis, platelets act as “immune switches,” regulating immune balance through their core mechanism: Platelets, which have both hemostatic and immune functions, interact with monocytes via the CD47 molecule on their surface and the corresponding receptor on monocytes. This interaction activates the AKT/mTOR-glycolytic signaling pathway within monocytes, driving epigenetic remodeling and immune reprogramming. Under physiological conditions, sufficient platelets can limit monocyte Toll-like receptor (TLR) responses through this pathway, maintaining immune tolerance. However, during sepsis-induced thrombocytopenia, this regulatory pathway fails, and the suppression of monocyte TLR responses is lifted. Monocytes transition from an immune-tolerant state to an overactive immune response, ultimately triggering systemic excessive inflammation and immune dysfunction.

Platelets are not merely pro-inflammatory effector cells; they function as “immune switches” with immune regulatory plasticity. Platelets participate in the precise regulation of inflammation and the maintenance of immune homeostasis. This “homeostasis regulation” function holds crucial pathophysiological significance throughout the sepsis course (64). Recent studies have confirmed that the CD47 molecule, highly expressed on the platelet surface, interacts specifically with the homologous CD47 molecule on monocytes. This interaction activates the AKT/mTOR signaling pathway in monocytes, driving glycolytic metabolic reprogramming, leading to epigenetic remodeling and immune phenotype switching in monocytes (64) (see **Figure 4**).

In physiological homeostasis or the early regulatory phase of sepsis, the continuous activation of this pathway effectively inhibits monocyte Toll-like receptor (TLR)-mediated pro-inflammatory signaling, enabling monocytes to maintain an immune-tolerant phenotype and preventing indiscriminate inflammatory activation. This ensures “precise modulation” of the inflammatory response—preserving the body’s defense against pathogens while preventing excessive activation of the systemic inflammatory response syndrome (SIRS) (64). However, when thrombocytopenia occurs during the progression of sepsis, this immune homeostasis regulation network becomes dysfunctional. A reduction in platelet count leads to weakened interaction between platelets and monocytes, disrupting AKT/mTOR-glycolytic pathway activation, inhibiting monocyte epigenetic remodeling and immune reprogramming, and relieving TLR signaling inhibition. As a result, monocytes shift to an overactive pro-inflammatory response phenotype, leading to massive cytokine release and even triggering a cytokine storm, characteristic of sepsis, which exacerbates multi-organ dysfunction (64, 130).

The clinical significance of this mechanism is crucial: sepsis-associated thrombocytopenia is not merely an abnormal platelet count but reflects the loss of platelet-mediated immune homeostasis regulation pathways (64, 130). This explains why patients with sepsis and thrombocytopenia often experience more severe inflammatory disorders and worse outcomes—platelet depletion not only directly impairs hemostatic function but also disrupts the regulatory balance of the immune network (43). In clinical treatment decisions, this mechanism suggests that interventions for patients with very low platelet counts must balance the dual effects—benefit-risk considerations. Restoring platelet counts may help restore immune homeostasis through CD47 signaling, but may also exacerbate immune thrombosis risks due to activation state differences of infused platelets. If left untreated, immune homeostasis regulation deficiency and bleeding risk may persist, leading to uncontrolled inflammation (131, 132). This complex pathophysiological feature further highlights platelets as a core “immune-coagulation integration regulatory hub” (115, 133).

### 3.4 Functional phenotype switching of platelets during disease progression

During sepsis progression, imbalance and dynamic shifts among the three major platelet functional phenotypes—the adhesive–aggregatory phenotype, inflammatory secretory phenotype, and procoagulant phenotype—directly determine the transition between their protective and deleterious effects. Notably, these phenotypic changes often precede platelet count decline and therefore may have important early warning value (8, 15). In the resting state, platelets primarily maintain hemostatic homeostasis, with low responsiveness of the ADP–P2Y axis, stable distribution of receptors such as GPVI and GPIbα, and baseline levels of adhesion and immune interaction, without a clear functional phenotype bias (114, 134). Under low-grade inflammation, corresponding to the early anti-infective stage, TLR signaling activates only a subset of platelets into a defensive hyperreactive state, dominated by the adhesive–aggregatory and inflammatory secretory phenotypes. The former promotes physical barrier formation through integrin αIIbβ3 activation, whereas the latter recruits immune cells by releasing mediators such as PF4 and IL-8, together facilitating pathogen clearance and endothelial homeostasis (115, 135). Under high-intensity inflammation, as in the cytokine storm stage, sustained Syk–NF-κB activation induces excessive secretion and receptor exhaustion, driving platelets toward an exhausted phenotype characterized by reduced CD41a and CD9 expression, decreased PAC-1 binding, and weakened responses to agonists such as ADP and collagen, ultimately impairing both hemostatic and immunoregulatory functions. At the same time, the proportion of the procoagulant phenotype increases markedly, with enhanced phosphatidylserine (PS) exposure and ANO6 channel activation accelerating thrombin generation and ultimately leading to microcirculatory dysfunction and organ injury (136, 137).

Platelet subpopulations from different origins exhibit distinct thresholds for inflammatory signaling. For example, lung-derived platelets express higher levels of immune-related receptors and show greater responsiveness. This feature further amplifies functional heterogeneity and may provide potential targets for stratified intervention (9). Assessment of functional indicators such as CD62P, PAC-1, and the proportion of platelet–neutrophil complexes (PNCs) may help define the current platelet phenotype and distinguish defensive activation from pathological injury (138).

## 4 Sepsis-related platelet abnormalities: mechanisms and bedside interpretation

Sepsis is a systemic inflammatory response syndrome triggered by infection (114), and its pathological core involves the bidirectional dysregulation of immune activation and coagulation systems (10, 61), often accompanied by multi-organ dysfunction risk (115, 126, 139). In this pathological context, sepsis-related platelet abnormalities are not simply “decreased counts” but represent a complex pathological manifestation of disturbances in platelet number, function, and temporal dynamics under an immune-coagulation microenvironment imbalance (64, 113, 140). Therefore, a deeper understanding of platelet abnormalities in sepsis, extending from basic pathological mechanisms to an actionable multidimensional assessment system, will provide theoretical reference and practical evidence for subsequent mechanistic studies and precise interventions, integrating clinical observable dynamic trends with specific pathologies (117).

### 4.1 Clinical value and limitations of platelet count: the discrepancy between quantity and function

Sepsis-related thrombocytopenia carries both anti-infection protection and inflammation-induced damage attributes, and intervention strategies should not solely rely on platelet count (115, 141). Studies have shown that 44.7% of sepsis patients experience thrombocytopenia, and the extent of platelet count reduction is significantly correlated with the severity of multiple organ dysfunction syndrome (MODS) and mortality (142). Some retrospective studies suggest that platelet count has a nonlinear relationship with prognosis; within a certain range, platelet counts are more likely to show protective effects, but a single static threshold cannot support refined decision-making (141, 143).

Traditionally, platelet transfusions or platelet-stimulation drugs are used in clinical practice, but the evidence is uncertain: on one hand, transfusion effects are unstable and may not improve prognosis in patients with varying severity; on the other hand, preventive transfusions in severe thrombocytopenia cases are unnecessary and may exacerbate inflammation, trigger immune imbalance, and lead to ineffective transfusion due to antibody-mediated responses (144). This does not imply that platelets are unimportant but highlights the dilemma of “untiered, untimed, and unpositioned” decision-making—identical low platelet counts may correspond to completely different leading mechanisms and risk trajectories (145, 146).

Pathophysiologically, platelets are both “functional markers” of immune defense and “drivers” of disease progression (8, 141).In sepsis, platelets participate in immune thrombosis and cell aggregate formation, and peripheral blood count dynamics can reflect infection-related consumption. However, in the context of an inflammatory storm, their immune regulatory imbalance can amplify thrombo-inflammation, destroy the endothelial barrier, impede microcirculation, and promote organ dysfunction (126, 147). At the same time, prolonged low platelet levels damage hemostatic function and increase the risk of intracranial and gastrointestinal bleeding (142). This dissociation between quantity and function urgently requires the establishment of a multidimensional stratified assessment system to achieve precise mechanistic judgment and treatment decisions (115, 143).

### 4.2 Pathological mechanisms of thrombocytopenia

The dissociation between platelet number and function and the clinical controversy over interventions are fundamentally attributed to the high heterogeneity of the pathological mechanisms underlying sepsis-associated thrombocytopenia (10, 145). A deeper exploration of the mechanisms reveals that this abnormality is caused by multiple interrelated pathological pathways—coagulation activation-mediated consumption, inflammation-driven reduced production, immune regulatory disruption leading to increased clearance, and abnormal distribution. The intrinsic logic and regulatory networks of these mechanisms will be gradually clarified through stratified analysis (146, 148, 149).

#### 4.2.1 Coagulation activation and consumptive reduction: DIC/SIC, immune thrombosis, and platelet consumption

In sepsis, the coagulation system and inflammatory response form a tightly intertwined bidirectional regulatory network. Disseminated intravascular coagulation (DIC) is a typical pathological manifestation of this network imbalance and one of the key pathophysiological mechanisms in sepsis with thrombocytopenia (TCP) (114, 126, 150). The core pathological feature of DIC is the excessive generation of thrombin, which leads to systemic microthrombosis formation and massive platelet consumption. This process is significantly correlated with patient mortality. Pathogen-derived PAMPs can directly trigger platelet activation, promote platelet-neutrophil complex (PNC) formation, and release pro-inflammatory factors, further aggravating coagulation dysfunction. Simultaneously, the deposition of fibrin-platelet thrombi within the microcirculation accelerates platelet consumption, leading to a progressive decline in peripheral platelet count (11, 50, 129, 151).

Importantly, coagulation activation-mediated platelet consumption does not occur in isolation, but acts synergistically at an early stage with immune clearance and abnormal distribution. On the one hand, proinflammatory mediators such as IL-1β released from PNCs can activate the complement system and accelerate immune-mediated platelet destruction (11). On the other hand, coagulation activation-induced endothelial injury enhances splenic platelet adhesion and sequestration, further reducing the circulating platelet pool and forming an early synergistic consumption network linking coagulation activation, immune clearance, and splenic sequestration (9). In addition, platelets can form platelet–leukocyte aggregates (PLAs), particularly with neutrophils, which further accelerate platelet consumption through immune amplification (129). Clinical studies have shown that circulating PLA levels are significantly elevated in patients with sepsis, and that PLA-derived proinflammatory mediators such as IL-1β can establish a thrombo-inflammatory positive feedback loop. Experimental studies further suggest that inhibiting PLA formation significantly improves survival in animal models of sepsis, highlighting this cooperative node as a potential target for early intervention (11, 135). At the same time, abnormal complement activation and vascular endothelial injury further amplify platelet destruction and sequestration, thereby sustaining pathological injury and reinforcing the dominant role of early platelet consumption (10).

#### 4.2.2 Reduced production: inflammation-related bone marrow suppression and hematopoietic signaling interference

**Figure 5:**
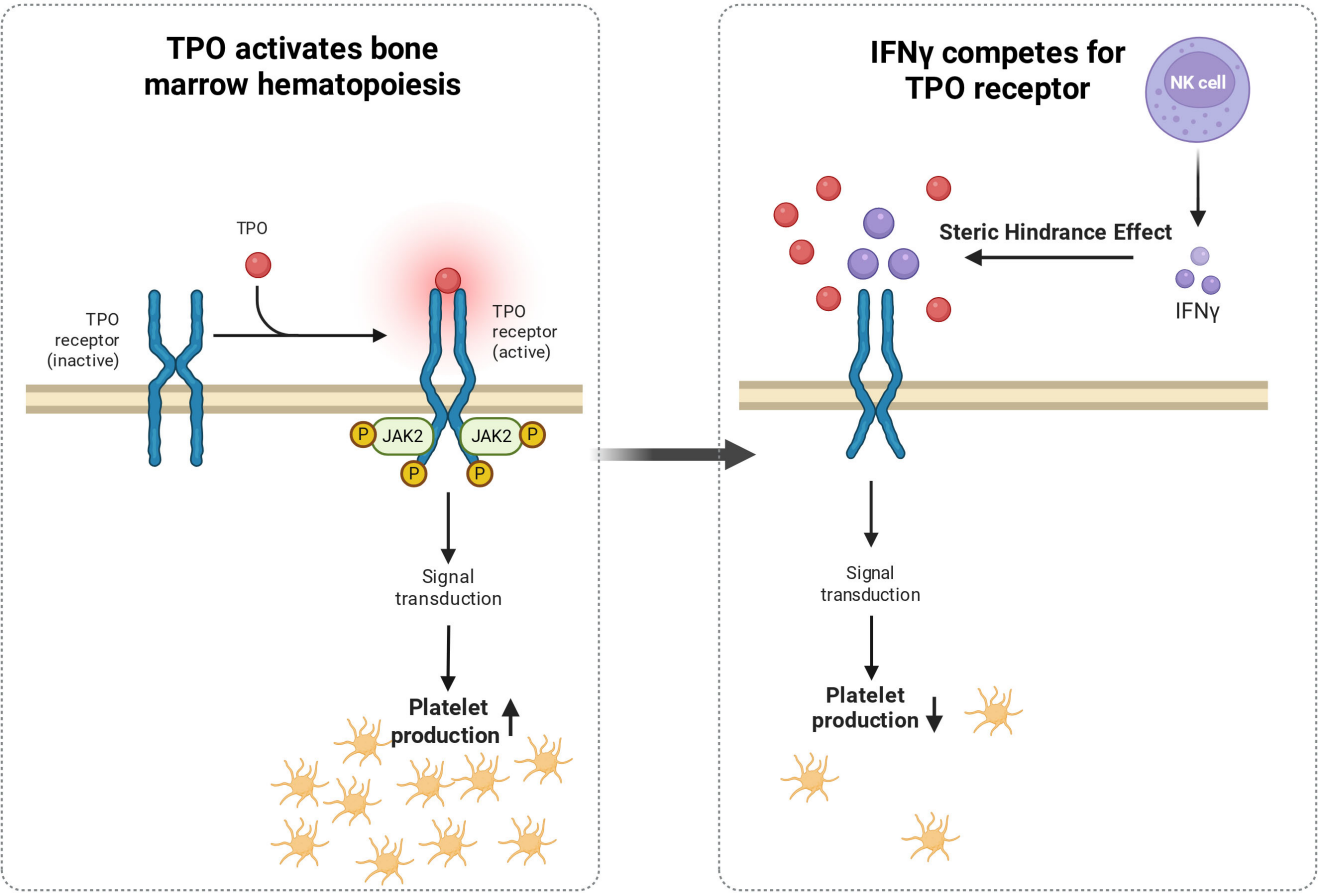
Inflammatory cytokines inhibit bone marrow platelet production. Left Panel: Thrombopoietin (TPO) binds to the TPO receptor on the surface of bone marrow cells, causing a conformational change and activation of the receptor. This activation initiates the JAK2 signaling pathway, which drives hematopoiesis in the bone marrow and promotes platelet production. Right Panel: Inflammatory cytokine IFN-γ competes for binding to the TPO receptor through steric hindrance, preventing normal TPO-receptor interaction. This weakens TPO-mediated signal transduction, ultimately reducing platelet production in the bone marrow. This illustrates the molecular mechanism of platelet production reduction in the inflammatory state.

Bone marrow suppression is one of the key pathogenic mechanisms of sepsis-associated thrombocytopenia (SAT) (9, 146). During sepsis, immune system activation leads to significantly increased levels of inflammatory cytokines such as TNF-α and IL-6: on one hand, these cytokines inhibit megakaryocyte differentiation and maturation via interference with the Mitogen-Activated Protein Kinase (MAPK) pathway, and on the other hand, they activate the Nuclear Factor κB (NF-κB) pathway, inducing megakaryocyte apoptosis. This ultimately reduces platelet production, and clinical evidence confirms a significant negative correlation between the levels of these cytokines and platelet count in patients (152–154). Additionally, high concentrations of Interferon-γ (IFN-γ) can competitively activate the Janus Kinase 2-Signal Transducer and Activator of Transcription (JAK2-STAT) pathway, antagonizing the thrombopoietin receptor TPO-R-mediated hematopoietic signaling by inhibiting JAK2 pathway activation, as shown in **Figure 5**, although its specific role in sepsis requires further exploration. Future studies may help to explore this pathway (155, 156).

In addition to classical inflammatory cytokines, increased phosphorylation of Bruton’s tyrosine kinase (BTK) in macrophages during sepsis drives M1-type macrophages to release IL-1β and TNF-α, forming a “inflammatory amplification-bone marrow suppression” vicious cycle. Inhibiting BTK can alleviate this process (146). Persistent inflammation also triggers endoplasmic reticulum stress in the bone marrow microenvironment, interfering with megakaryocyte differentiation and promoting apoptosis, while also inhibiting hematopoietic stem cell function (157–159). Notably, emerging mechanisms such as ferroptosis and mitochondrial dysfunction in hematopoietic regulation are gradually being revealed, suggesting that SAT pathogenesis has complex, multidimensional features (157).These emerging pathways may also act synergistically with inflammation-mediated bone marrow suppression, further weakening platelet production. Clinically, patients in the middle and late stages of sepsis often present with persistently low platelet counts and poor recovery, largely because inadequate bone marrow production cannot compensate for ongoing consumption and immune clearance. This mechanism also provides a rationale for the use of thrombopoietic agents in selected patients during the later stages of sepsis (9, 145).

#### 4.2.3 Immune-mediated clearance: complement, desialylation, and PRR-related apoptosis

Immune-mediated platelet destruction is also a key pathological mechanism contributing to sepsis-associated thrombocytopenia, involving multiple pathways such as complement system activation, abnormal glycosylation, and pattern recognition receptor (PRR)-mediated apoptosis (160, 161).Importantly, this process does not act in isolation. Rather, it extends throughout the course of sepsis and acts in concert with coagulation activation and bone marrow suppression, amplifying platelet consumption in the early stage and aggravating the failure of compensatory platelet production in the middle and late stages. Overactivation of the complement system not only generates pro-inflammatory molecules like C5a, amplifying the inflammatory response, but its terminal product C5b-9 (membrane attack complex) can also directly assemble on the platelet membrane, causing platelet lysis through pore formation. Targeting the complement-coagulation cross-talk pathway has been shown to alleviate sepsis-related pathological damage (162).As noted above, complement activation can be triggered by proinflammatory mediators released from early platelet–neutrophil complexes (PNCs) and platelet–leukocyte aggregates (PLAs), thereby forming a cooperative network of coagulation activation, complement amplification, and platelet consumption that accelerates early thrombocytopenia. In the middle and late stages, persistent complement activation may further damage newly generated platelets, weaken compensation by bone marrow production, and act synergistically with bone marrow suppression (163–165).

**Figure 6:**
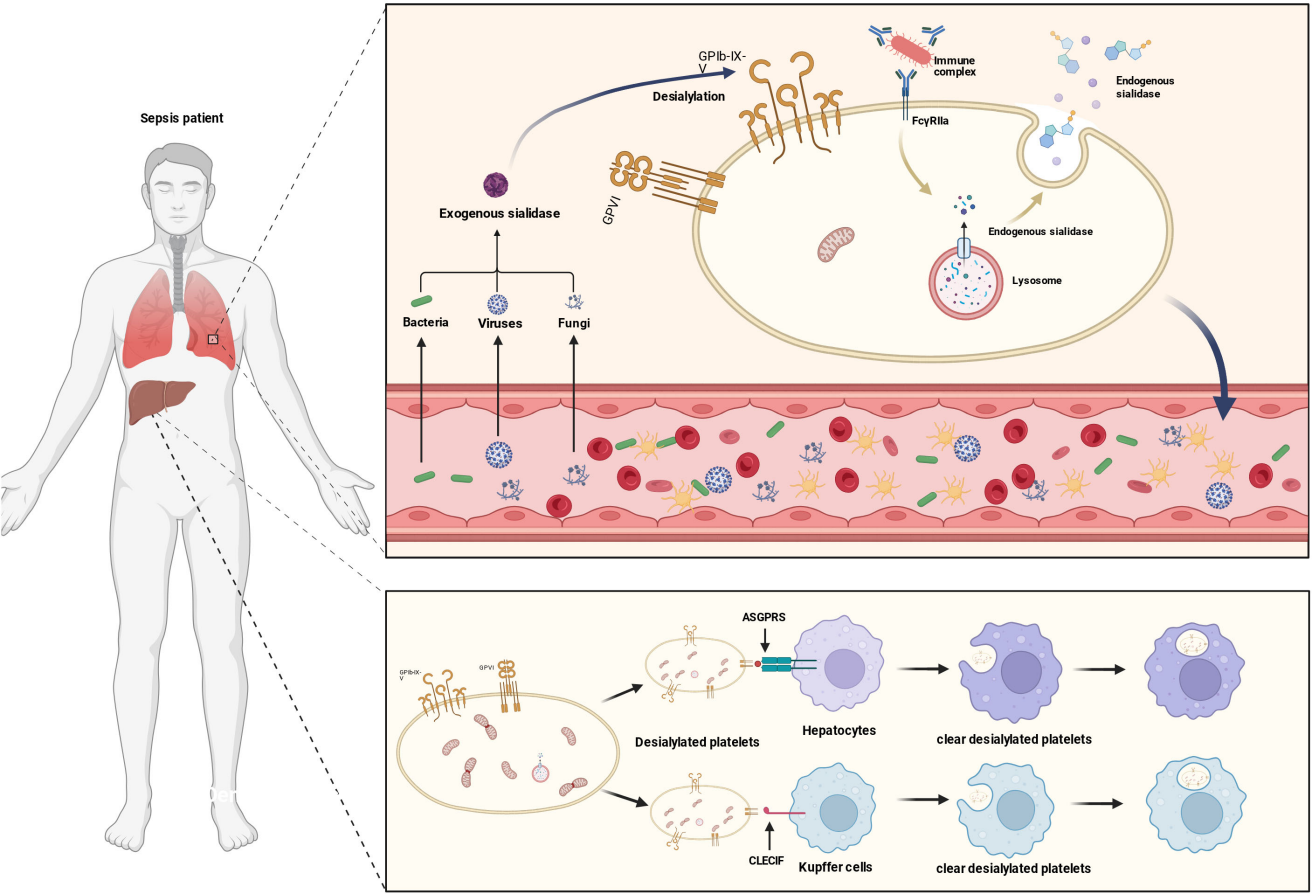
Hepatic Kupffer cell clearance of desialylated platelets. Left Panel: Systemic background in septic patients. Right Panel, Upper Module: In sepsis, exogenous neuraminidase released by pathogens (bacteria, viruses, fungi) or endogenous neuraminidase released from lysosomes removes sialic acid modifications from the platelet surface, resulting in desialylation of platelets and exposure of specific surface structures. Right Panel, Lower Module: Kupffer cells in the liver recognize desialylated platelets through specific receptors such as ASGPR (Asialoglycoprotein Receptor) and C-Type Lectin Receptor IF (CLECIF), leading to their phagocytosis and clearance, ultimately resulting in a decrease in platelet count in septic patients.

Additionally, desialylation of platelet surface glycoproteins is a significant mechanism accelerating platelet clearance. In sepsis, bacterial-derived neuraminidase induces the removal of sialic acid residues from platelets, exposing terminal glycans such as β-galactoside (β-Gal), which are specifically recognized by the Ashwell-Morell receptor (AMR) on liver cells. This triggers phagocytosis and clearance of platelets by Kupffer cells (liver macrophages), as depicted in **Figure 6**. Clinical studies have confirmed that the proportion of desialylated platelets in peripheral blood correlates negatively with platelet count in sepsis patients (160, 162). This mechanism also closely interacts with coagulation activation and inflammatory responses. Endothelial injury induced by coagulation activation may increase platelet contact with hepatocytes, whereas inflammatory mediators can upregulate AMR expression on Kupffer cells, further enhancing phagocytic clearance (166).Some studies suggest that in immune thrombocytopenic purpura (ITP) patients, antibodies can promote the translocation of platelet surface neuraminidase 1 (NEU1) through an FcγRIIa-dependent signaling pathway, leading to desialylation (167). This suggests that this receptor signaling pathway may be related to endogenous neuraminidase activation in sepsis, and that persistent immune-inflammatory dysregulation may further amplify desialylation-mediated platelet clearance.

Another critical mechanism is the abnormal activation of the TLR7 signaling pathway. In the pathological microenvironment of sepsis, TLR7 triggers downstream cascades via a MyD88-dependent pathway, resulting in platelet mitochondrial damage and apoptosis. In experiments, TLR7 agonist Resiquimod induces a thrombocytopenia phenotype in mice, and the physical interaction and tyrosine phosphorylation regulation of TLR7 by the epidermal growth factor receptor (EGFR) are key to the activation of this pathway (168–170).TLR7 signaling can be triggered directly by PAMPs and further amplified by complement activation products. TLR7-mediated platelet apoptosis not only increases platelet consumption, but may also promote the release of proinflammatory mediators, thereby further aggravating inflammation and bone marrow suppression and forming a cooperative cycle of immune clearance, inflammatory amplification, and reduced platelet production. These immune-mediated destructive mechanisms are interconnected, collectively exacerbating abnormal platelet consumption in sepsis. Blocking AMR recognition, inhibiting desialylation, or targeting the TLR7/EGFR signaling pathway may become potential therapeutic strategies to alleviate sepsis-associated thrombocytopenia (169, 171).

#### 4.2.4 Abnormal distribution: spleen sequestration and peripheral blood platelet decrease

In sepsis, the decrease in platelet count is closely related to abnormal platelet distribution (10, 114, 145).The core mechanism is the dual regulatory effect of the spleen on platelet production and sequestration. This process extends throughout the course of sepsis and acts as an auxiliary mechanism that cooperates with consumption, immune clearance, and impaired production to aggravate thrombocytopenia. Studies have found that abnormal platelet distribution is closely associated with dynamic changes in spleen function. During sepsis, the spleen exhibits a significant dual regulatory effect on platelet production and sequestration (9, 145). On the one hand, the spleen becomes the primary site of platelet production: Sepsis-induced adrenergic signaling activation mediates the migration of megakaryocyte-erythroid progenitors (MEPs) from the bone marrow to the spleen microenvironment, where they differentiate into fully mature megakaryocytes under the influence of cytokines such as IL-3. These spleen-derived megakaryocytes produce specific platelets with high expression of CD40 ligand, which have strong immune regulatory functions. Animal experiments have confirmed that transfusing these platelets can significantly enhance the immune response in sepsis models (9).This production pathway represents a compensatory response to insufficient bone marrow thrombopoiesis. It becomes particularly important in the middle and late stages of sepsis, when bone marrow suppression worsens. However, this compensation is often offset by ongoing consumption, immune clearance, and splenic sequestration, and therefore fails to effectively reverse thrombocytopenia (145, 154).

On the other hand, spleen sequestration is a key factor in the reduction of circulating platelets: During sepsis, the spleen microenvironment undergoes inflammatory remodeling, and the adhesion between platelets and splenic sinusoidal endothelial cells is enhanced, leading to abnormal retention (sequestration) of a large number of platelets in the spleen. This pathological sequestration, combined with increased platelet consumption, exacerbates the decline in peripheral platelet count. In the early stage, this process is mainly associated with coagulation activation and complement-mediated platelet activation, as activated platelets are more prone to adhere to splenic sinusoidal endothelium. In the middle and late stages, it acts synergistically with inadequate compensation from bone marrow suppression, leading to a further decrease in circulating platelet levels (172, 173).Clinical studies have found that platelet distribution abnormalities related to spleen sequestration are an independent predictor of poor prognosis in sepsis patients (10, 114, 145). A major reason is that splenic sequestration not only reduces circulating platelets, but also promotes platelet dysfunction, as retained platelets are more likely to undergo activation or apoptosis, thereby further aggravating immune–coagulation dysregulation (27).Moreover, sepsis-induced activation of spleen immune cells and cytokine network dysregulation may further amplify this dual effect, increasing the complexity of thrombocytopenia (9, 174).

#### 4.2.5 Clinical interpretation of the pathological mechanism network

The pathological mechanisms underlying sepsis-associated thrombocytopenia form a continuous pathophysiological network, with inflammation–coagulation activation as the central trigger, consumption, immune clearance, and splenic sequestration as early synergistic events, bone marrow suppression as the dominant mechanism in the middle and late stages, and compensatory splenic platelet production as an auxiliary regulatory response. The interplay among these mechanisms determines the dynamic changes in platelet count and functional status, and also provides a basis for stratified intervention (9, 141).

From a clinical perspective, dynamic changes in platelet count may directly reflect activation of this pathological network. The dominant mechanisms, corresponding clinical indicators, and potential intervention directions of sepsis-associated thrombocytopenia are summarized in **Table 3**. In early sepsis (24–72 h after infection), a rapid decline in platelet count accompanied by coagulation abnormalities, such as prolonged prothrombin time (PT) and elevated D-dimer, suggests that coagulation activation-driven consumption, complement activation, and splenic sequestration are the dominant processes. At this stage, intervention should focus on infection control and suppression of the coagulation–inflammation network, while avoiding indiscriminate platelet transfusion (142, 175, 176). In the middle and late stages of sepsis (>72 h after infection), persistently low platelet counts with poor recovery, together with sustained elevation of inflammatory mediators, suggest that bone marrow suppression has become the dominant mechanism and is acting in concert with ongoing immune clearance and splenic sequestration. In this context, thrombopoietic agents such as thrombopoietin receptor agonists (TPO-RAs) may be considered on the basis of infection control, while the degree of splenic sequestration should also be evaluated to avoid ineffective transfusion (146, 177). Linking this mechanism network to clinical interpretation may help overcome the previously noted dilemmas of non-stratified, mistimed, and non-targeted decision-making, thereby enabling mechanism-oriented precision intervention. This translational value is a major clinical reason for further clarifying the pathological network of platelet abnormalities in sepsis (113, 115).

**Table 3:** Dominant mechanisms, clinical features, and potential intervention directions of sepsis-associated thrombocytopenia.

Dominant mechanism	Clinical indicator features	Potential intervention direction
Coagulation activation-mediated consumption	Rapid decline in platelet count (PLT) + elevated D-dimer + prolonged prothrombin time (PT)	Infection control plus coordinated anti-inflammatory and anticoagulant intervention
Bone marrow suppression	Persistently low PLT + no increase in mean platelet volume (MPV) or immature platelet fraction (IPF) + elevated interleukin-6 (IL-6)	Thrombopoiesis-stimulating therapy, such as thrombopoietin receptor agonists (TPO-RAs)
Immune-mediated clearance	Declining PLT + increased proportion of desialylated platelets	Avoid indiscriminate platelet transfusion; explore neuraminidase inhibition
Splenic sequestration	Fluctuating PLT + splenomegaly	Evaluate splenic function and dynamically monitor platelet recovery

It should be emphasized that the current mechanism stratification based on disease course remains preliminary and represents only a rough stage-based framework with clear limitations. On the one hand, it does not adequately account for interindividual differences, such as underlying diseases, pathogen type, and immune status, all of which may substantially influence the timing and magnitude of dominant mechanisms (141, 178). On the other hand, existing stratification lacks sufficient support from large-scale clinical datasets, and no clear consensus has yet been reached regarding the quantitative relationships among interacting mechanisms or the differential efficacy of interventions across strata (10, 179). Therefore, more prospective, large-scale clinical studies are still needed, ideally integrating multicenter data with precise functional and mechanistic assays, to refine stratification criteria, define pathological patterns in different patient populations, and optimize intervention strategies. Such efforts may ultimately enable a transition from rough stage-based stratification to precise individualized intervention.

### 4.3 From platelet count monitoring to multivariable assessment

Solely relying on platelet count has significant limitations: count reflects only the end balance of “production and consumption” with a delayed response and cannot capture the hidden changes in platelet function and bone marrow dynamics in the early stages of sepsis. Therefore, sepsis prognosis evaluation should shift from a single static count to a dynamic, multidimensional assessment encompassing morphology and composite indicators.

#### 4.3.1 Early warning value of morphological parameters

Compared to platelet count, platelet morphological indices—mean platelet volume (MPV), platelet distribution width (PDW), and large platelet ratio (LPR)—are more sensitive in detecting early pathological changes (141, 180). Studies have shown that inflammatory storms (e.g., IL-6, TPO surge) stimulate megakaryocytes in the bone marrow to release larger, more metabolically active “immature platelets” (reticulated platelets) (9, 154, 181). These young platelets contain more α-granules and mRNA, with higher enzymatic activity and pro-thrombotic potential (141, 182). Therefore, increases in MPV and LPR not only indicate compensatory hematopoiesis in the bone marrow but may also suggest elevated thrombo-inflammatory risk. PDW widening, reflecting increased platelet volume heterogeneity, often occurs before a decrease in platelet count (141, 183–185). Moreover, the dynamic changes in immature platelet fraction (IPF) have been shown to be closely related to the progression of organ dysfunction (186), making it a sensitive indicator for assessing bone marrow recovery capacity (14).

#### 4.3.2 Composite indicators and combined assessment strategies

In addition to morphological parameters, novel composite indicators show predictive efficacy (143, 187). The platelet-to-lymphocyte ratio (PLR) reflects the balance between inflammation and immune status, with higher PLR associated with poor prognosis in severe patients (188). Functionally, decreased platelet aggregation rate has been confirmed to predict poor outcomes, possibly related to mitochondrial dysfunction induced by sepsis (reduced ATP synthesis, opening of Mitochondrial Permeability Transition Pore, mPTP) (14). It is worth noting that combining platelet-related parameters with classical biomarkers can further optimize risk stratification. For example, combining platelet count with procalcitonin (PCT) may help identify subgroups with immune homeostasis imbalance that traditional inflammatory markers cannot distinguish (189). The greater value of these combined indicators lies in helping to explain the clinical contradiction of “platelet count changes not synchronized with organ burden” (190).

The core value of these combined assessment strategies is to provide a mechanistic explanation for the clinical contradiction of “platelet count changes not synchronized with organ damage load” and further validate the need to move beyond single platelet count assessments in sepsis to a multidimensional, dynamic comprehensive judgment (143, 190).

#### 4.3.3 Sequential multi-parameter integration: a bedside-applicable clinical decision pathway

**Figure 7:**
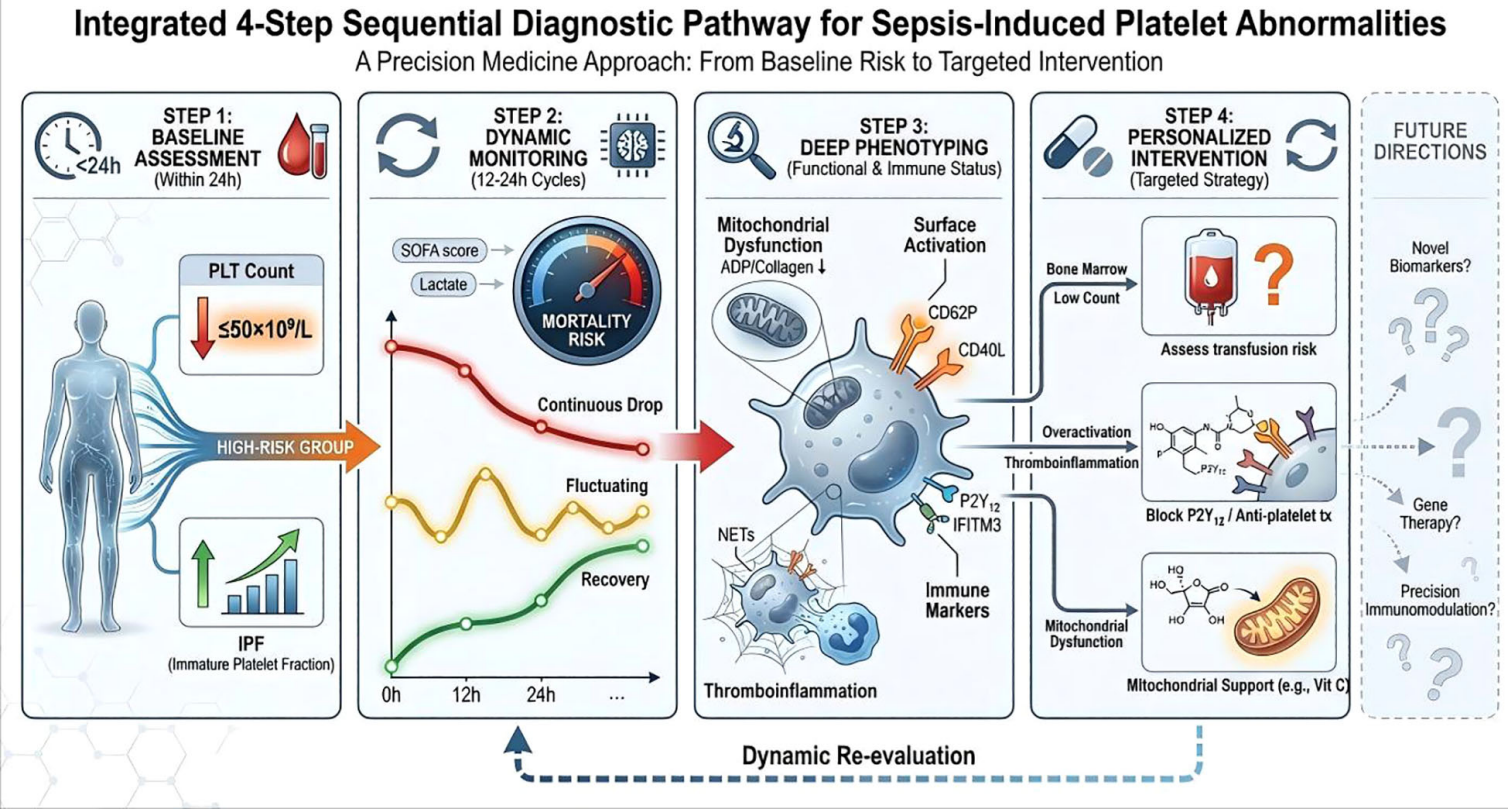
Sequential diagnostic workflow for sepsis-induced platelet abnormalities. A four-step workflow from baseline risk assessment to targeted intervention. High-risk patients are first identified by platelet count and IPF, followed by dynamic monitoring with platelet trajectories, SOFA score, and lactate. Functional and immune phenotyping is then used to define the dominant pathological state, including mitochondrial dysfunction, platelet activation, and thrombo-inflammatory features. Finally, individualized interventions are selected according to platelet phenotype and risk profile, with dynamic re-evaluation performed throughout the disease course. Future applications may include novel biomarkers, gene-based therapies, and precision immunomodulatory strategies.

Based on the pathological mechanism network outlined above and current clinical needs, we propose a four-step sequential decision pathway supported by available clinical evidence (**Figure 7**). This framework dynamically integrates platelet count, morphological parameters, functional phenotypes, and immune markers at the bedside to guide stratified assessment, risk updating, and individualized intervention.

First, baseline early warning assessment should be performed within 24 h of admission, including platelet count (PLT) and immature platelet fraction (IPF). A PLT ≤50 × 10^9/L indicates a high risk of 28-day mortality, whereas PLT <100 × 10^9/L warrants dynamic monitoring (191). An elevated IPF suggests active bone marrow compensation and is positively associated with disease severity and mortality. Patients with confirmed sepsis and either PLT ≤50 × 10^9/L or markedly elevated IPF should therefore be classified as high risk and enter intensive monitoring.

Second, in high-risk patients, PLT should be monitored every 12–24 h, and machine learning-based approaches may be used to assess dynamic platelet trajectories. For example, joint latent class models (JLCMs) can identify individualized trajectory patterns such as persistently declining, fluctuating, or recovering PLT profiles. A decline in PLT from ≥100 × 10^9/L to ≤50 × 10^9/L suggests worsening prognosis. At the same time, clinical parameters such as lactate, Sequential Organ Failure Assessment (SOFA) score, and Pediatric Index of Mortality-3 (PIM-3) in children can be incorporated into dynamic prediction models to update 28-day mortality risk in real time (191, 192).

Third, in high-risk patients or those with worsening PLT trajectories, deeper evaluation of functional and immune phenotypes should be considered. This may include assessment of ADP- or collagen-induced platelet aggregation, where reduced aggregation may indicate mitochondrial dysfunction and serve as an early predictor of mortality; platelet activation markers such as CD62P and CD40L; mitochondria-related molecules such as OPA1 and SIRT3; immunoregulatory markers including the P2Y12 receptor, IFITM3, and STK10; as well as platelet–neutrophil complexes (PNCs) and neutrophil extracellular traps (NETs). Together, these markers help define platelet functional state, activation level, and thrombo-inflammatory progression (14, 129).

Fourth, individualized intervention and dynamic feedback should be matched to the results of the preceding assessments. In patients with continuously declining PLT, elevated IPF, and functional suppression, impaired platelet production or increased consumption should be considered, and indications for platelet transfusion should be strictly evaluated, especially given the potential harm of transfusion in patients with activation-consumption phenotypes (114, 162). If platelet activation markers are markedly elevated and PNC levels increase, indiscriminate transfusion should be avoided, and targeted strategies such as the P2Y antagonist ticagrelor or anti-P-selectin therapy may be explored (126). When mitochondrial dysfunction appears to be the dominant feature, interventions such as vitamin C may be considered, based on clinical trial evidence, to improve microcirculation. Reassessment every 48 h is recommended to dynamically update both trajectory models and intervention strategies (190).

Notably, this pathway still has important limitations. It does not yet fully incorporate individual differences such as underlying diseases, immune status, and pathogen type, and some targeted interventions still lack robust evidence from large-scale clinical studies. Future prospective, multicenter investigations combined with precise detection technologies are therefore needed to refine the assessment criteria and quantitative thresholds for each step, validate the efficacy of targeted interventions, and optimize the overall decision pathway. Such efforts may help advance the evaluation and management of sepsis-related platelet abnormalities from rough stratification toward precise individualized intervention, thereby maximizing the translational value of multi-parameter integrated assessment (115, 193).

## 5 Stratified assessment

The significant feature of platelet abnormalities in sepsis is that a single platelet count indicator is insufficient to reflect the complexity of platelet function and its pathological origins. However, this challenge is not solely due to the lack of detection technologies but also results from the current detection information not being systematically integrated and interpreted in a stratified manner. In current clinical and research settings, platelet detection methods cover aspects such as quantity, morphology, reactivity, activation phenotype, and some immune-related characteristics, but these indicators often exist as “isolated values” and have not been incorporated into a unified stratification framework. There is no clear distinction in terms of the time sensitivity of different indicators, nor is there an understanding of their complementary roles in pathological mechanism interpretation.

Therefore, this section systematically reviews the types of information provided by different hierarchical indicators, their time sensitivity, and their potential roles in precise stratification. It aims to define the boundaries and complementary values of each indicator to lay the foundation for constructing a multi-parameter, mechanism-driven stratification system for sepsis-associated thrombocytopenia (SAT).

### 5.1 Quantity and time dimensions

Platelet count remains the most widely used, stable, and reproducible indicator of platelet assessment (145, 190, 194). However, count itself only reflects the net result of “production and consumption,” making it a typical “endpoint variable” with limited sensitivity to early pathological changes. A significant decrease in platelet count only occurs when the rate of platelet consumption exceeds the compensatory capacity of the bone marrow, making it difficult to capture the subtle pathological disturbances in the early stages of sepsis (141).

Compared to the absolute value of a single time point, the time dynamics of platelet count provide richer information, including the rate and magnitude of decline, the time window at which the lowest point occurs, whether there is a rebound after infection control, and the speed of that rebound (190). These dynamic features reflect the intensity of platelet consumption or immune-mediated clearance, the relationship with the inflammatory peak and organ damage progression, and the compensatory capacity of bone marrow hematopoiesis (114, 116, 141). While these do not directly point to specific mechanisms, they can serve as “trigger signals” for subsequent functional and mechanistic assessments. Additionally, combining count thresholds (mild: 101-140×10^9^/μL, moderate: 51-100×10^9^/μL, severe: 21-50×10^9^/μL, very severe: ≤20×10^9^/μL) can further refine risk stratification, providing a preliminary reference for clinical intervention priorities (176, 194, 195).

### 5.2 Morphological indicators

Platelet morphological parameters commonly reported in routine blood tests—mean platelet volume (MPV), platelet distribution width (PDW), large platelet ratio (LPR), and immature platelet fraction (IPF)—are often overlooked in clinical practice but have significant early informational value in the context of sepsis.

These indices mainly reflect two biological aspects: the reactivity of the generation end and the heterogeneity of peripheral platelet populations (105, 155). As previously mentioned, their core value lies in indicating that “the platelet population structure is changing.” In stratified assessments, this dimension can serve as “transitional information” that links changes in platelet count with functional remodeling, providing key clues to differentiate between “pure consumption” and “generation-function reconstruction.” Additionally, some studies have shown that abnormal morphological patterns are influenced by pathogen type, which may help distinguish platelet abnormalities associated with bacterial and viral infections (131, 141).Moreover, platelet size heterogeneity is closely related to platelet function, as larger platelets exhibit greater adhesive and thrombotic capacity, partly due to higher expression of GPVI and HLA-I.

### 5.3 Functional and activation phenotype detection

Platelet function testing is the closest current method to “functional typing” and is a core tool for addressing the clinical dilemma of “same platelet count, different risks” (15, 103). Emerging technologies such as mass cytometry and full-spectrum flow cytometry have further enhanced the depth and precision of platelet function and activation phenotype assessment.

Through flow cytometry or aggregation functional assays (e.g., Light Transmission Aggregometry, LTA), platelet reactivity and activation states can be assessed from multiple dimensions:Expression levels of surface activation markers (such as P-selectin/CD62P, activated αIIbβ3 integrin), which directly reflect the degree of platelet activation. Aggregation response strength to different agonists (e.g., ADP, collagen, thrombin), which can assess platelet actual hemostatic capacity. Expression or availability of key receptors (e.g., GPVI, GPIbα), which reveal platelet binding potential to ligands such as collagen and vWF (15, 30, 103).Mass cytometry allows simultaneous detection of over 20 markers at the single-platelet level, overcoming the parameter limitations of conventional flow cytometry. This approach can precisely identify functionally heterogeneous platelet subpopulations in sepsis and characterize differences in activation, secretion, and procoagulant phenotypes. For instance, during infection, a subpopulation with high baseline activation marker expression but reduced responsiveness to stimulation (“highly activated–functionally desensitized”) can be identified, providing refined evidence for functional typing (196–198).

These tests are not routinely performed at the bedside but are accessible in centers with appropriate facilities. Their core value lies in decoupling “platelet count” from “actual physiological function.” For example, some patients may have only a slight decrease in platelet count, but due to GPVI receptor shedding or mitochondrial dysfunction, their aggregation function may be significantly weakened, resulting in a higher bleeding risk compared to patients with lower platelet counts but normal function. Conversely, other patients may have low platelet counts but show high expression of P-selectin and enhanced aggregation, with the core risk shifting to immune thrombosis and microcirculatory dysfunction (14, 15). Functional and activation phenotype detection can identify whether platelets are in a high-reactivity, relatively resting, or exhausted-like low-reactivity state, thus providing mechanistic explanations for organ damage risk or bleeding tendencies (14, 15, 30).

### 5.4 Platelet-immune related indicators: from “hemostatic cells” to “immune nodes”

With the deepening understanding of platelet immune functions, some detection indicators have started to move beyond traditional hemostatic functions. Platelet-derived extracellular vesicles (PDEVs, also referred to as platelet microparticles, PMPs) and platelet–immune interaction markers have become important supplements for stratifying platelet abnormalities in sepsis, directly reflecting their involvement in the immune-inflammatory network. These indicators include the platelet-leukocyte aggregate (PLA/PNC) ratio, platelet-derived microparticle (PMP) levels, and the expression of immune-related molecules on the platelet surface (such as CD40L, FcγRIIa, CD47) (4, 114, 133, 141, 199).

PMPs are membrane-bound vesicles released upon platelet activation and serve as core mediators of platelet paracrine function. They are enriched in procoagulant molecules such as phosphatidylserine, inflammatory mediators including IL-1β and TNF-α, and adhesion molecules. In sepsis, PMP levels are markedly elevated and closely associated with microcirculatory dysfunction and multi-organ injury (200). PLA/PNC ratios and CD40L expression can be quantified using flow cytometry, while PMPs can be assessed through microfluidic platforms or flow-based approaches. Although some of these assays remain in research or semi-research stages, they provide important mechanistic insights. Their primary significance lies in embedding platelet abnormalities directly within the immune–inflammatory network and paracrine regulation, helping to differentiate whether platelet count reduction is merely a pathological consequence or whether platelets themselves act as drivers of inflammatory amplification (113). Moreover, PMPs may serve as novel biomarkers of thrombo-inflammatory progression in sepsis, providing a more specific paracrine dimension for stratified assessment (201, 202).

### 5.5 Single-cell and transcriptomic technologies: platelet heterogeneity and molecular mechanisms

Advanced technologies such as platelet RNA sequencing, single-cell RNA sequencing, and mass cytometry enable in-depth analysis of platelet heterogeneity, molecular characteristics, and pathological pathways, providing molecular-level evidence for precise stratification of platelet abnormalities in sepsis. These approaches represent a major advancement in the field (203).

Although platelets are anucleate, they contain abundant mRNAs, non-coding RNAs, and circular RNAs. Under pathological conditions, their transcriptome can exhibit alterations in 10 to over 1,000 transcripts. Platelet RNA sequencing pipelines (e.g., thromboSeq) require as little as 500 pg RNA to complete the full analysis and can identify key molecular pathways and biomarkers associated with platelet abnormalities in sepsis. Examples include detecting mitochondrial dysfunction and immune activation–related differentially expressed genes, constructing platelet transcriptomic scores linked to prognosis, and clarifying platelet participation in signaling pathways such as NF-κB and JAK2–STAT, thereby providing molecular evidence for mechanistic stratification (52).Single-cell RNA sequencing can circumvent leukocyte contamination and precisely characterize transcriptomic profiles of functionally distinct platelet subpopulations in sepsis, including hyperreactive and exhausted platelets, revealing molecular heterogeneity within the platelet population (204). Combined with mass cytometry–based protein profiling, these approaches allow multi-dimensional integration of transcriptomic and proteomic data, delineating molecular regulatory networks underlying platelet abnormalities in sepsis (196). Furthermore, these technologies can dissect the origins of platelet heterogeneity at the single-cell level, distinguishing subpopulation changes driven by abnormal bone marrow production from those caused by peripheral activation or clearance, thereby providing precise molecular targets for intervention (114, 205, 206). Currently, platelet RNA sequencing and single-cell approaches remain primarily research tools, but their capacity to resolve platelet abnormalities at the molecular level lays the groundwork for extending stratified assessment from functional phenotyping to molecular subtyping (204, 207).

Overall, current platelet assessment methods already encompass multiple layers, including quantitative dynamics, morphology, functional activation, immune–paracrine status, and transcriptomics. Emerging tools such as platelet-derived extracellular vesicles, single-cell technologies, and multi-omics platforms further enhance the capacity to dissect platelet abnormalities in sepsis. The key challenge is not merely the lack of detection tools, but the absence of a stratified framework that integrates and interprets these data in a pathophysiologically coherent manner. In clinical practice, conventional indices are often interpreted in isolation, and the findings from advanced technologies have yet to be effectively translated into stratified clinical assessment, leaving the information chain incomplete.

Future precision stratification will not necessarily require entirely new technologies, but rather integration and translation of existing accessible indicators and advanced technological findings. By combining multi-dimensional analyses, distinct platelet abnormality subtypes can be identified, including consumption-dominant, production-deficient, immune-mediated destruction, and hyperreactive activated platelets, while molecular features allow even finer stratification (10). The core of stratified assessment is the logical linkage of indicators rather than simple accumulation of data. The ultimate goal is to convert dispersed numerical, functional, and molecular information into precise pathological subtyping. On this basis, subsequent therapeutic decisions and study designs can achieve true mechanism-aligned, risk-weighted intervention, rather than repeatedly adjusting treatment based solely on platelet count, thereby providing a scientific foundation for precision intervention in sepsis-associated platelet abnormalities. Additionally, efforts are needed to standardize and translate mass cytometry, platelet RNA sequencing, and related technologies into clinical practice, addressing sample requirements, assay costs, and feasibility, so that molecular-level stratified assessment can be progressively applied at the bedside (52).

## 6 Treatment strategies and research directions

The core challenge in managing sepsis-associated platelet abnormalities lies in their heterogeneity and the dual effects of interventions. The same platelet count may correspond to distinct pathological subtypes, including insufficient production, immune-mediated clearance, or high consumption, and any intervention must balance correcting bleeding risk against potentially triggering thrombo-inflammatory amplification. Therefore, treatment should not be simplified to merely “increasing platelet count,” but should focus on shortening the window of vulnerability, reducing organ perfusion injury, and preventing bleeding complications. All strategies must be integrated with upstream infection control and organ support, as interventions targeting platelets alone are unlikely to alter core outcomes. In this section, we discuss strategies based on stratified platelet subtypes, emphasizing optimal patient selection, timing, and risk–benefit considerations, to strengthen precision matching between subtype and intervention.

### 6.1 Platelet transfusion: risk and dynamic assessment

Platelet transfusion provides direct compensation for hemostatic capacity, but in the inflammatory microenvironment of sepsis, transfused platelets may participate in immunothrombosis and microcirculatory regulation via adhesion, aggregation, and mediator release, producing highly variable effects. Therefore, transfusion decisions must be guided by subtype stratification, clinical context, and dynamic assessment, avoiding mechanical supplementation based solely on numeric thresholds. The core principle is that subtype-adapted transfusion should take precedence over count-based thresholds.

#### 6.1.1 Applicable scenarios and subtype-specific matching

Therapeutic transfusion is indicated only for patients with active bleeding (e.g., intracranial or gastrointestinal hemorrhage) or those at high bleeding risk (PLT ≤20 × 10^9/L with coagulation abnormalities), with the goal of rapidly restoring hemostatic capacity rather than normalizing platelet count (208). It is particularly suitable for patients in the middle to late stages with “pure consumption” (coagulation activation–dominant) or “splenic sequestration” subtypes, as platelet loss in these subtypes is primarily physical, and transfusion efficiently restores hemostatic function (195).

Prophylactic transfusion requires integration of multiple factors and consideration of platelet subtype. Key considerations include: 1. Risk of invasive procedures, such as mechanical ventilation, continuous renal replacement therapy (CRRT), or surgery. 2. Coagulation status, with indicators such as elevated D-dimer or prolonged PT suggesting high consumption. 3.Subtype characteristics: Patients with immune-mediated clearance subtypes (e.g., desialylation- or complement-driven) or PNC/NET-driven high-consumption subtypes should generally avoid indiscriminate transfusion, as transfused platelets may accelerate immune clearance or thrombo-inflammatory coupling, worsening the disease. In contrast, patients with production-deficient subtypes who require high-risk procedures may cautiously receive small-volume transfusions (148, 209, 210).

#### 6.1.2 Key points for clinical implementation

The core principles for clinical transfusion are timed, minimal, and dynamically assessed. Small, single-dose transfusions should be preferred, with dynamic monitoring of PLT recovery, bleeding control, and lactate/microcirculatory indicators to avoid repeated large-volume transfusions (211). Clinicians should remain alert to potential risks under strong inflammatory conditions: transfused platelets may promote platelet–neutrophil aggregate (PNC) formation and microvascular thrombosis via CD40L- or P-selectin–mediated interactions. Repeated transfusions may also induce antibody-mediated ineffective transfusion. For patients with immune-mediated clearance subtypes, if transfusion is necessary, concurrent evaluation of immunosuppressive interventions (e.g., short-term corticosteroids) may help reduce platelet destruction (164, 212, 213).

### 6.2 Anticoagulant and antiplatelet therapy: timing and intensity based on functional phenotypes

The central challenge of anticoagulant and antiplatelet therapy in sepsis is the “mistimed intervention risk”: premature inhibition may impair platelet-mediated anti-infective defense, whereas delayed intervention may fail to block organ damage driven by immunothrombosis. Decisions must therefore be guided by platelet subtype and functional phenotype to avoid “intervention without stratification.”

#### 6.2.1 Stratified Application of Anticoagulant Therapy

Routine prophylaxis: Low molecular weight heparin (LMWH) is commonly used for venous thromboembolism (VTE) prevention in sepsis patients without high bleeding risk and who do not exhibit a severe consumption-dominant phenotype (214).

Enhanced anticoagulation: Recommended only for patients with confirmed coagulation activation–driven consumption and no active bleeding. Strict monitoring of platelet count, fibrinogen, and bleeding signs is required. Contraindications include PLT ≤50 × 10^9/L, severe coagulation factor deficiency (PT >1.5 × normal), or active bleeding (215, 216). For patients with immune-thrombosis–coupled phenotypes (elevated PNCs/NETs), enhanced anticoagulation may be combined with anti-NET therapy (e.g., DNase) to increase specificity of intervention (217).

#### 6.2.2 Precision Selection of Antiplatelet Therapy

Antiplatelet therapy is not a standard treatment for sepsis and should be considered only in two scenarios:1.Patients with underlying cardiovascular or cerebrovascular disease (e.g., coronary artery disease, prior stroke).2.Patients whose functional phenotype indicates a hyperreactive activated subtype (e.g., high P-selectin expression, increased aggregation, elevated PNC proportion) (10, 218). Clinically, antiplatelet therapy should be avoided in early sepsis, when infection is uncontrolled and platelets are in a defensive activation phase. In immunothrombosis-dominant stages (elevated PNCs and active NET formation) without high bleeding risk, P2Y_12_ antagonists may be cautiously considered. Patients with exhausted or production-deficient subtypes should receive antiplatelet therapy with caution, as it may further impair hemostasis and increase bleeding risk. In the absence of functional phenotype assessment, empirical intensification of antiplatelet therapy is unlikely to provide stable benefit and may instead result in an unfavorable risk–benefit balance (10, 214).

### 6.3 Thrombopoiesis-stimulating therapy

#### 6.3.1 Indications and subtype matching

Clear indications for thrombopoiesis-stimulating therapy include:1.Stratified assessment indicating bone marrow suppression–dominant subtype (e.g., no increase in MPV/IPF, persistently elevated inflammatory mediators, and absence of high-consumption evidence such as normal D-dimer).2.Persistently low platelet counts (PLT ≤50 × 10^9/L) with high bleeding risk.3.Need to shorten the duration of thrombocytopenia to reduce bleeding or procedural risk. The core principle for subtype matching is that production-deficient subtypes preferentially receive thrombopoietin receptor agonists (TPO-RAs). For immune clearance subtypes, because platelet destruction mechanisms are not resolved, stimulation of production alone is often insufficient and should be combined with immunomodulatory therapy. High-consumption subtypes require prior control of infection and coagulation activation, otherwise stimulation cannot compensate for ongoing loss (209, 219, 220).

#### 6.3.2 Assessment of efficacy and ineffective scenarios

Efficacy should be evaluated through dynamic monitoring of platelet recovery, with a doubling of PLT within 5–7 days considered indicative of effective response, and trends should align with clinical improvement. If platelet counts rise but lactate levels remain elevated or microcirculatory dysfunction persists, this suggests that newly produced platelets are still participating in immunothrombosis, and intervention focus may need adjustment (e.g., intensifying anti-inflammatory or anticoagulant therapy).Certain scenarios predict limited efficacy:1.Uncontrolled cytokine storm in high-consumption subtypes.2.Immune clearance–dominant subtypes. In these cases, thrombopoiesis stimulation alone is unlikely to improve clinical outcomes and may even produce the paradox of rising platelet counts with worsening organ function. Coordination with infection control and anti-inflammatory therapy is therefore essential (221–223).

### 6.4 Mechanistic targeted strategies: potential targets and evidence stage

Nanodelivery, MicroRNA (miRNA), inflammasome inhibition, and natural active molecules are all cutting-edge, mechanism-driven strategies. However, the evidence for these approaches remains primarily in the preclinical/basic stages. Future trials will need to focus more on subtype stratification and selection of appropriate time windows. We summarize potential therapeutic approaches, their mechanisms of action, and the challenges associated with them in **Table 4**.

**Table 4:** Treatment strategies and challenges for sepsis-associated thrombocytopenia.

Strategy/Method	Target action	Research stage	Potential challenges/Limitations	References
Targeting Platelet Desialylation Mechanisms	Reducing platelet clearance by using sialidase inhibitors or targeting the desialylated glycoprotein receptor on hepatic macrophages.	Preclinical studies, animal models	Lack of target specificity, efficacy and safety need further validation.	(148, 160, 171, 224, 225)
Inhibition of Platelet Pyroptosis	Blocking key pathways of platelet pyroptosis to alleviate inflammation and improve thrombocytopenia.	Preclinical studies, animal models	The molecular mechanisms of pyroptosis are complex, and effective intervention of pyroptosis pathways requires further exploration. Dynamic immune state changes in different stages of sepsis necessitate precise timing for intervention.	(8, 46, 226–228)
Targeting Bruton’s Tyrosine Kinase (BTK) Signaling Pathway	Using BTK inhibitors to reduce macrophage inflammation, decrease platelet destruction or consumption, and improve thrombocytopenia.	Preclinical studies, clinical trials	Safety and off-target effects of BTK inhibitors; immune suppression exists, and clinical use must weigh anti-inflammatory benefits against immune suppression risks.	(64, 146, 229–231)
Modulating Platelet Immune Function	Developing therapies to restore or modulate platelet immune function, breaking the pro-inflammatory interaction between platelets and immune cells, and improving immune homeostasis.	Proof of concept, *in vitro* studies	How to precisely modulate platelet immune function without compromising immune defense.	(64, 115, 117, 132, 140, 177)
Nanotechnology Drug Delivery System	Designing multifunctional nanoparticles that combine anti-inflammatory and platelet-generating therapies to improve treatment efficacy and targeting.	*In vitro* and animal model experiments	Lack of target specificity, limited delivery efficiency, and optimization of nanoparticle design is still required.	(232–235, 249)
Thrombopoietin Receptor Agonists (TPO-RAs)	Promoting platelet production, increasing platelet count, and improving sepsis-related coagulation dysfunction.	Clinical stage	The efficacy for sepsis patients is not fully established, more research specifically for sepsis is needed.	(146, 209, 236–238)
NLRP3 Inflammasome Inhibitors	Alleviating the systemic inflammatory response of sepsis, improving platelet consumption, and mitigating disseminated intravascular coagulation (DIC)-related pathology.	*In vitro* and animal model experiments	Low bioavailability, inadequate delivery efficiency.	(35, 140, 146, 239–242)
MicroRNA (miRNA) Therapy	Modulating miRNA expression to regulate platelet activation, inflammatory cytokine release, or cell apoptosis to improve platelet function and overall prognosis.	Proof of concept, *in vitro* studies	Insufficient mechanistic research, further studies are needed to explore the role of miRNA in sepsis and its specific targets.	(64, 243–246)

## 7 Discussion

Sepsis, as a systemic inflammatory and coagulation disorder syndrome triggered by infection, remains a clinical challenge due to its high incidence and mortality rates. Platelets, as a core hub in the “inflammation-coagulation-immune” network, play a dual role as both “protectors” and “drivers” in disease progression. They restrict pathogen spread in early stages through immune recognition and defense mechanisms, yet may exacerbate immunothrombosis and multi-organ injury if overactivated or functionally exhausted. Precision management of platelet abnormalities in sepsis thus requires a shift from mere platelet count correction toward mechanism–phenotype–time window matching, representing a key transition from empirical intervention to stratified precision care.

Traditional count-based assessments are no longer sufficient, highlighting the need for multidimensional and dynamic platelet evaluation strategies. Current practice, however, faces multiple challenges. Technically, advanced tools such as mass cytometry and single-cell RNA sequencing are complex, require large sample volumes, and have long processing times, limiting their bedside applicability (203). Standardized protocols for microfluidics and nanoparticle tracking analysis are lacking, reducing inter-laboratory comparability (247). Economically, high costs of advanced equipment and reagents limit multicenter implementation, particularly in resource-constrained settings. Clinically, many physicians remain familiar only with conventional hemostatic functions, with limited understanding of functional phenotypes or molecular mechanisms, and electronic medical record systems often lack modules to integrate multidimensional indices for dynamic stratified decision-making (115, 248).

Emerging therapeutic strategies also require cautious evaluation. Nanodelivery systems offer targeting advantages but face barriers including insufficient specificity, uncertain *in vivo* safety, and scalability issues, and remain largely at the *in vitro* or animal model stage (249). miRNA-based therapies show potential to modulate platelet activation and inflammatory responses but are limited by off-target effects, low bioavailability, and uncertain stability within the complex septic microenvironment (128). Mechanism-targeted drugs, such as BTK inhibitors or pyroptosis pathway antagonists, may increase infection risk due to immunosuppression, and lack optimized dosing and timing for different sepsis subtypes, making indiscriminate use potentially harmful.

Future research should focus on three main directions to advance precision and standardization in managing sepsis-related platelet abnormalities:1.Technology translation: Develop bedside-adapted functional assays that are low-cost, rapid, and operationally simple. Establish standardized protocols for detecting platelet functional phenotypes and subtype markers (e.g., desialylated platelets, IPF) to overcome barriers to clinical implementation (250).2.Clinical studies: Conduct multicenter, prospective studies to validate subtype stratification and intervention efficacy. Determine prognostic value and intervention thresholds for consumption-dominant, production-deficient, and immune clearance subtypes, and evaluate safety and effectiveness of subtype-specific therapies (e.g., TPO-RAs for production-deficient, BTK inhibitors for macrophage-activated subtypes). This will generate high-quality clinical evidence.3.Decision-support tools: Integrate multidimensional assessments into intelligent stratification frameworks. Combine platelet counts, morphological parameters, functional phenotypes, immune markers, clinical features, and disease time windows to develop smart decision-support systems embedded within clinical platforms, reducing operational complexity for clinicians (143).

Additionally, further exploration of dynamic platelet signaling networks (e.g., TLR–NF-κB, CD47–AKT/mTOR) is needed to deepen understanding of the origins of platelet heterogeneity and mechanisms of phenotype switching, thereby identifying precise molecular targets for intervention. With coordinated progress in technology translation, clinical validation, and decision-support integration, precision subtyping and targeted management of platelet abnormalities in sepsis may become achievable, offering new avenues to improve patient survival and prognosis.
