# Supplementary material for: Stratified assessment of platelets in sepsis: from dynamic counts to functional phenotypes
Source: Front Immunol. 2026 Apr 10;17:1783001. doi: 10.3389/fimmu.2026.1783001 (PMC13105893; doi:10.3389/fimmu.2026.1783001)
Supplement: Supplementary file 1 [file Table1.docx]

Supplementary Material

**Table 3** Functional Classification of Platelet Receptors Relevant to Sepsis and Immunothrombosis

| Receptor Function | Category | Receptor Molecule | Function |
| --- | --- | --- | --- |
| Adhesion and aggregation receptors | Integrin family | αIIbβ3 (GPIIb/IIIa) | Core aggregation receptor; binds fibrinogen and mediates platelet–platelet crosslinking. |
|  |  | α2β1 | Collagen receptor; cooperates with GPVI to enhance collagen-dependent platelet activation. |
|  |  | α5β1 | Binds fibronectin. |
|  |  | α6β1 | Binds laminin. |
|  |  | αvβ3 | Binds vitronectin; vitamin K–dependent protein receptor. |
|  | LRR family | GPIbα (GPIb–IX–V complex) | Core adhesion receptor; binds von Willebrand factor (vWF) under high shear stress to initiate platelet adhesion. |
|  | Others | Layilin | Hyaluronan receptor; may mediate leukocyte interactions or exert inhibitory effects on adhesion. |
| Signal transduction receptors | GPCRs (ADP/TxA₂) | P2Y1 / P2Y12 | Mediate platelet shape change (P2Y1) and thrombus growth and stabilization (P2Y12), respectively. |
|  |  | Thromboxane receptor TPα / TPβ (CD233) | Responds to thromboxane A₂; induces potent vasoconstriction and platelet aggregation. |
|  |  | GPR31 | Participates in platelet activation via Rap1 and p38 signaling pathways. |
|  |  | GPR56 | Adhesion GPCR responsive to collagen and shear stress; activates Gα13 signaling to regulate platelet shape change. |
|  | GPCRs (thrombin) | PAR1 (primary) | Thrombin activates platelets predominantly through PAR1-mediated signaling. |
|  |  | PAR3 | Cofactor facilitating thrombin localization to promote PAR4 cleavage. |
|  |  | PAR4 (secondary) | Low-affinity receptor mediating sustained signaling and thrombus stabilization. |
|  | Ion channels | P2X1 | ATP-gated calcium channel facilitating rapid platelet shape change. |
|  | ITAM / collagen receptor | GPVI | Major collagen signaling receptor; also recognizes fibrin and triggers potent platelet activation. |
| Immune-related receptors | Pattern recognition receptors | TLRs | Recognize pathogen- and damage-associated molecular patterns, initiating immune activation. |
|  |  | NLRs | Cytosolic sensors involved in inflammasome activation and inflammatory signaling. |
|  |  | CLEC-2 | Central hub of thrombo-inflammation; binds podoplanin to drive immunothrombosis and tumor metastasis. |
|  | Fc receptor | FcγRIIa (CD32a) | Binds IgG immune complexes; mediates antibody-dependent cellular cytotoxicity (ADCC) and heparin-induced thrombocytopenia (HIT). |
|  | Cytokine / chemokine receptors | IL-1R1 / IL-1R8 | Mediate IL-1 signaling to drive inflammatory cascades; IL-1R8 exerts negative regulatory effects. |
|  |  | CCR7, CXCR4 | Sense chemokines and guide platelet trafficking to lymph nodes or inflammatory sites. |
|  |  | ACKR3 | Atypical chemokine receptor that scavenges chemokines and contributes to inflammation resolution. |
|  | Immune checkpoint / inhibitory receptors | Siglec-9 | Binds sialic acids; inhibits platelet activation and neutrophil extracellular trap (NET) formation. |
|  |  | PECAM-1 (CD31) | Contains ITIM motifs; maintains vascular integrity and suppresses excessive immune activation. |
|  |  | CD40 | Expressed upon activation; mediates immune signal transduction. |
|  | Costimulatory molecules / ligands | CD40L (CD154) | Expressed on activated platelets; participates in immune regulation. |
|  |  | P-selectin (CD62P) | Upregulated in inflammatory conditions; mediates cell adhesion, platelet–leukocyte interactions, and thrombosis. |
|  |  | MHC class I (HLA) | Antigen presentation; presents exogenous antigenic peptides to CD8⁺ T cells, linking innate and adaptive immunity. |
|  |  | CD63 | Key surface regulatory molecule involved in membrane protein trafficking, platelet activation, and intercellular communication (exosomes); serves as an activation marker influencing cell adhesion. |
|  |  | ICAM-2 | Expressed on platelets; mediates immune cell (e.g., T cell) activation and migration, particularly during inflammation. |
|  |  | JAM-A | Expressed on platelets and endothelial cells; regulates leukocyte transmigration, vascular inflammation, and thrombosis. |
|  |  | CD44 | Involved in cell adhesion and inflammation; in cancer, associated with metastasis and anti-inflammatory mechanisms via binding to activated platelets. |

**Table 4.** Functional Composition and Biological Roles of Platelet α-Granules, Dense Granules, and Lysosomes. Some molecules may participate in both inflammatory regulation and tissue repair depending on the pathological context.

**the functional classification is provided for conceptual illustration and does not imply mutual exclusivity.**

| Secretory Granule | Functional Role | Functional Molecule | Mechanism of Action |
| --- | --- | --- | --- |
| α-granules | Hemostasis and coagulation–related molecules | Fibrinogen | Endocytosed or synthesized by megakaryocytes and stored in α-granules; upon release, participates in fibrin network formation and stabilizes platelet plugs. |
|  |  | von Willebrand factor (vWF) and vWF propeptide (vWFpp) | Stored in eccentric nanodomains of α-granules; differentially secreted upon activation to promote platelet adhesion to injured vascular walls. |
|  |  | β-thromboglobulin (BTG) | Released as a marker of platelet activation; associated with cerebral microbleeds and ischemic injury. |
|  |  | Coagulation factor Xa (FXa) | Stored in α-granules; released FXa directly participates in coagulation pathways, reducing bleeding risk. |
|  | Inflammation and immune regulation | CD40 ligand (CD40L) | Secreted from α-granules; mediates platelet–leukocyte interactions and activates immune cells. |
|  |  | Platelet factor 4 (PF4/CXCL4) | Chemokine that modulates neutrophil activation and neutrophil extracellular trap (NET) formation, amplifying inflammatory responses. |
|  |  | Antimicrobial proteins/peptides | Directly kill pathogens or regulate NET formation; key mediators of innate immunity and immunothrombosis. |
|  |  | Chemokines (e.g., RANTES/CCL5) | Act on leukocytes to promote platelet–leukocyte aggregation and inflammatory signaling, particularly monocyte and lymphocyte recruitment. |
|  |  | Transforming growth factor-β1 (TGF-β1) | Regulates myeloid-derived suppressor cell (MDSC) function and modulates immune responses. |
|  |  | Tissue inhibitor of metalloproteinases-1 (TIMP-1) | Inhibits matrix metalloproteinases, regulating extracellular matrix remodeling, hemostasis, tissue repair, and inflammation. |
|  | Tissue repair and regeneration | Transforming growth factor-β1 (TGF-β1) | Regulates cell proliferation and differentiation; plays key roles in wound healing and immune suppression. |
|  |  | Thrombospondin-1 (TSP-1) | Major α-granule component; involved in tissue repair and non-alcoholic steatohepatitis via modulation of angiogenesis and cell migration. |
|  |  | SPARC (secreted protein acidic and rich in cysteine) | Regulates extracellular matrix assembly. |
|  |  | Osteopontin (OPN) | Promotes angiogenesis and tissue remodeling; upregulated during wound healing. |
|  |  | Angiopoietins (ANGPTs) | Promote angiogenesis and tissue remodeling during repair processes. |
|  |  | Stromal cell–derived factor-1 (SDF-1/CXCL12) | Stored in α-granules; released to support stem cell homing and tissue regeneration. |
|  |  | Platelet-derived growth factor (PDGF) | Released upon platelet activation; promotes cell proliferation, angiogenesis, and tissue repair. |
|  |  | Vascular endothelial growth factor (VEGF) | Released upon activation; drives angiogenesis and tissue regeneration. |
|  | Granule-specific enzymes and regulatory proteins | VPS33B/VPS16B complex | Mediates α-granule biogenesis; defects cause α-granule deficiency (ARC syndrome). |
|  |  | NBEAL2 | Maintains retention of α-granule contents; mutations cause Gray platelet syndrome (GPS). |
|  |  | α-synuclein | Regulates SNARE-complex assembly and granule secretion, platelet aggregation, and thrombus stability, thereby maintaining normal hemostasis. |
| Dense granules | Nucleotides | ATP | Serves as an energy source supporting platelet activation and cellular responses. |
|  |  | ADP | Amplifies platelet activation and aggregation by binding to platelet P2Y receptors. |
|  | Amines | Serotonin (5-HT) | Released upon activation; promotes vasoconstriction, inflammation, and thrombosis. |
|  | Ions | Ca²⁺, Zn²⁺ | Act as electrolytes and signaling mediators, regulating intracellular signaling, enzyme activity, and platelet shape change. |
| Lysosomes | Enzymes | Proteases, lipases, glycosidases, and other hydrolases | Mediate protein, lipid, and polysaccharide degradation. |
|  | Growth/nutritional factors | Various growth factors | Promote cell proliferation and differentiation. |
|  |  | Repair-related factors | Support tissue repair mechanisms and both innate and adaptive immunity. |
|  | Signaling molecules | Prostaglandin E₂ (PGE₂) | Modulates inflammatory responses and promotes pro-resolving mediator synthesis. |
|  |  | Transforming growth factor-β (TGF-β) | Suppresses pro-inflammatory cytokines and participates in inflammation resolution and tissue repair. |
|  |  | C-type lectin-like receptor-2 (CLEC-2) | Influences macrophage polarization and T-cell differentiation, modulating immune responses. |
|  | Lipids and lipid mediators | Lysophosphatidic acid (LPA) | Released upon platelet activation; induces cell shape change and invasiveness. |
|  |  | Sphingosine-1-phosphate (S1P) | Secreted lipid mediator regulating inflammation and thrombosis. |
|  |  | Lysophosphatidylserine (LysoPS) / Lysophosphatidylinositol (LPI) | Increased in the platelet lipidome; involved in diverse pathophysiological functions. |
|  |  | Bis(monoacylglycero)phosphate (BMP) | Lysosomal lipid that stimulates lipid-degrading enzymes; altered in disease states and may influence the cellular microenvironment. |
